# The toxicity of nanoparticles and their interaction with cells: an *in vitro* metabolomic perspective

**DOI:** 10.1039/d2na00534d

**Published:** 2023-01-30

**Authors:** Mohammad Awashra, Piotr Młynarz

**Affiliations:** a Department of Chemistry and Materials Science, School of Chemical Engineering, Aalto University 02150 Espoo Finland mohammad.awashra@aalto.fi; b Department of Biochemistry, Molecular Biology and Biotechnology, Faculty of Chemistry, Wroclaw University of Science and Technology Wroclaw Poland piotr.mlynarz@pwr.edu.pl

## Abstract

Nowadays, nanomaterials (NMs) are widely present in daily life due to their significant benefits, as demonstrated by their application in many fields such as biomedicine, engineering, food, cosmetics, sensing, and energy. However, the increasing production of NMs multiplies the chances of their release into the surrounding environment, making human exposure to NMs inevitable. Currently, nanotoxicology is a crucial field, which focuses on studying the toxicity of NMs. The toxicity or effects of nanoparticles (NPs) on the environment and humans can be preliminary assessed *in vitro* using cell models. However, the conventional cytotoxicity assays, such as the MTT assay, have some drawbacks including the possibility of interference with the studied NPs. Therefore, it is necessary to employ more advanced techniques that provide high throughput analysis and avoid interferences. In this case, metabolomics is one of the most powerful bioanalytical strategies to assess the toxicity of different materials. By measuring the metabolic change upon the introduction of a stimulus, this technique can reveal the molecular information of the toxicity induced by NPs. This provides the opportunity to design novel and efficient nanodrugs and minimizes the risks of NPs used in industry and other fields. Initially, this review summarizes the ways that NPs and cells interact and the NP parameters that play a role in this interaction, and then the assessment of these interactions using conventional assays and the challenges encountered are discussed. Subsequently, in the main part, we introduce the recent studies employing metabolomics for the assessment of these interactions *in vitro*.

## Abstract 2

The interaction of nanoparticles (NPs) with cells depends on their physicochemical properties and can lead to cytotoxic events. Metabolomics reveals the molecular mechanisms of this nanotoxicity and avoids the misleading interferences with NPs.

## Graphical abstract

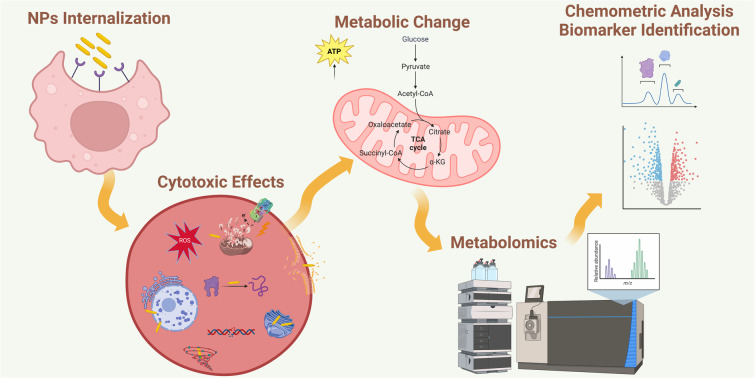

## 1. Introduction

Nanomaterials are defined as materials with at least one dimension smaller than 100 nm,^1^ while nanotechnology is defined as the understanding and manipulation of matter at dimensions in the range of 1 to 100 nm, where unique phenomena enable novel applications.^2^ Nanotechnology introduces many potential health, environmental, and industrial benefits^3,4^ and its applications are widespread in daily life, transforming society.^5^ For example, its applications in the food industry range from agriculture^6^ to food processing and packaging.^7^ Furthermore, nanotechnology is applied in drug delivery,^8^ imaging, diagnostics, cosmetics,^9^ clothing,^10^ transportation,^11^ biofuels,^12^ and biosensors.^13^ Therefore, human exposure to nanomaterials (NMs) nowadays is highly probable. Depending on the type of product in which nanoparticles (NPs) are used, exposure may occur through inhalation, dermal, and oral pathways. Among them, inhalation is considered as the most significant exposure route for airborne NPs.^14^

Due to their high surface to volume ratio, high reactivity, and tunable characteristic properties, NMs exhibit great benefits such as enhanced targeting and imaging techniques.^15^ However, NMs may also cause some potential risks to human health and the environment.^16^ Given that human and environmental exposure to NMs are inevitable, nanotoxicity research is attracting increasing attention.^17^ In the last decade, the number of research studies on the toxicity of different types of NMs has increased dramatically. NMs may affect human health in several ways such as inflammation^18^ and heart problems.^19,20^ Thus, to understand the mechanisms of these effects, more investigations in the nanotoxicology field are necessary at the cellular and sub-cellular levels. The scope of nanotoxicity depends on many parameters that are related to the NM itself such as its size, shape, chemical composition, and coating^21^ or the exposed cell type or tissue.^22^

When exposed to NPs, the cell can be affected *via* several routes, including a decrease in cell viability and proliferation,^23^ inflammatory response, production of cytokines,^24,25^ oxidative stress,^26,27^ generation of reactive oxygen species (ROS),^28–30^ cell membrane damage,^29^ mitochondrial damage,^28,31^ cell cycle dysregulation,^27^ DNA damage,^32^ genotoxicity,^24^ lipid peroxidation, changes in cell morphology,^33^ apoptosis^29^ or necrosis,^25^ and metabolic changes.^30^ To study the cytotoxicity of NPs, many conventional assays and biomarkers are used. For example, the cell viability and proliferation can be investigated using tetrazolium-based assays such as MTT,^34^ MTS,^35^ and WST-1.^36^ Alternatively, the cell inflammatory response can be investigated by measuring inflammatory biomarkers, such as IL-8, IL-6, and tumor necrosis factor, using ELISA.^37^ Moreover, for cell membrane integrity, lactate dehydrogenase (LDH) and trypan blue exclusion assays can be used,^37^ and for cell metabolism, the Alamar Blue assay is frequently used.^38^ However, although these assays afford general information about the cytotoxicity of NPs, they do not give molecular information about the mechanism of their cytotoxicity.^38–41^ Moreover, NPs can interfere with the conventional assays, and thus the use of more than one assay is important. In general, most of the studies on the cytotoxicity of NPs mainly use the conventional (phenotypic) tests and assays. Alternatively, some studies used other techniques based on the change in epigenome, transcriptome, proteome, or metabolome (omics techniques) induced by NPs.^39^ These techniques are beneficial to study the effect of NPs on cells at the molecular level and explain the results of conventional essays. Among them, metabolomics is one of the most powerful bioanalytical strategies, enabling a picture of the metabolites of an organism in the course of a biological process to be obtained, which is the omics technique of interest in this review.^40,41^ The introduction of NPs in a cell line may cause a change in the levels of certain metabolites, which may give a clue on their effect on cells. During the past decade, many *in vitro* studies have used metabolomics to investigate the cytotoxicity of NPs on different cell lines.

In this review, the ways NPs and cells interact and the effects of the NP parameters on their interaction are discussed, followed by an overview of the cytotoxicity of different NPs in *in vitro* models, focusing on the use of metabolomics as a tool to identify the mechanisms and molecular information of their cytotoxicity.

## 2. Cellular uptake of NPs

The cytotoxic effects of NPs usually originate from their presence inside cells.^42^ However, many applications of NMs in biomedicine require their entry in the cell to achieve their goal. Therefore, to further understand the cytotoxicity mechanisms of NPs on the cell and its metabolism, it is important to first understand the cellular uptake mechanisms of NPs. This will also aid the design of environmentally safer NMs with enhanced cellular targeting and uptake properties for therapeutic purposes.^43^

When immersed in a biological fluid, NPs are exposed to a different medium than that employed for their synthesis. This will force the NPs to interact with the surrounding medium, which may alter their physical and chemical properties.^44^ To stabilize themselves, NPs tend “to catch” the surrounding biomolecules (proteins, lipids, *etc.*) and form a biomolecular corona or protein corona (in case they are surrounded by proteins only), which may alter their identity.^45^

NPs may be taken up by the cell in an energy-independent process, such as simple diffusion or translocation. However, most NP uptake pathways are energy dependent *via* endocytosis. Endocytosis is the formation of vesicles from the cell plasma membrane to take up substances such as particles, nutrients, and dead cells from the extracellular to the intracellular environment.^46^ Endocytosis is described in two categories, *i.e.*, phagocytosis and pinocytosis.

### 2.1 Phagocytosis

**Fig. 1:**
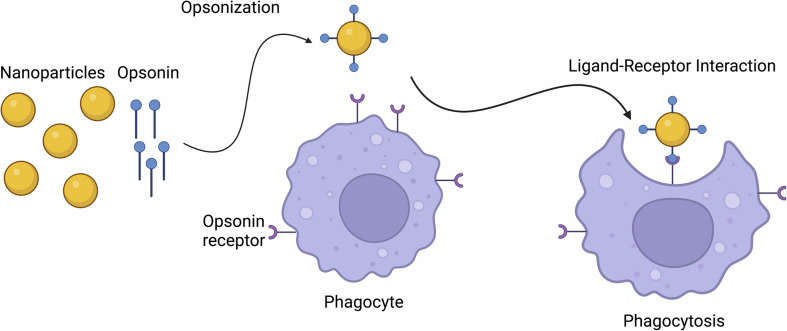
Nanoparticle internalization *via* phagocytoses.

Phagocytosis is the cellular uptake of particulates (0.5–10 μm) in the plasma-membrane envelope. It is known as a host defence mechanism, engulfing and internalizing cargos such as particles, dead cells, and cell debris.^43,47^ This mechanism is a ligand-induced process, where NPs are engulfed by adsorbing opsonins, followed by their interaction with complement receptors on the cell surface (see Fig. 1).^48^

### 2.2 Pinocytosis

Pinocytosis is the cellular uptake of extracellular fluids and dissolved solutes.^49^ It can be divided into macropinocytosis, clathrin- and caveolae-independent endocytosis, and receptor-mediated endocytosis. The latter is classified as clathrin-dependent endocytosis and caveolae-dependent endocytosis based on the proteins involved in the pathway.^50^

#### 2.2.1 Macropinocytosis

**Fig. 2:**
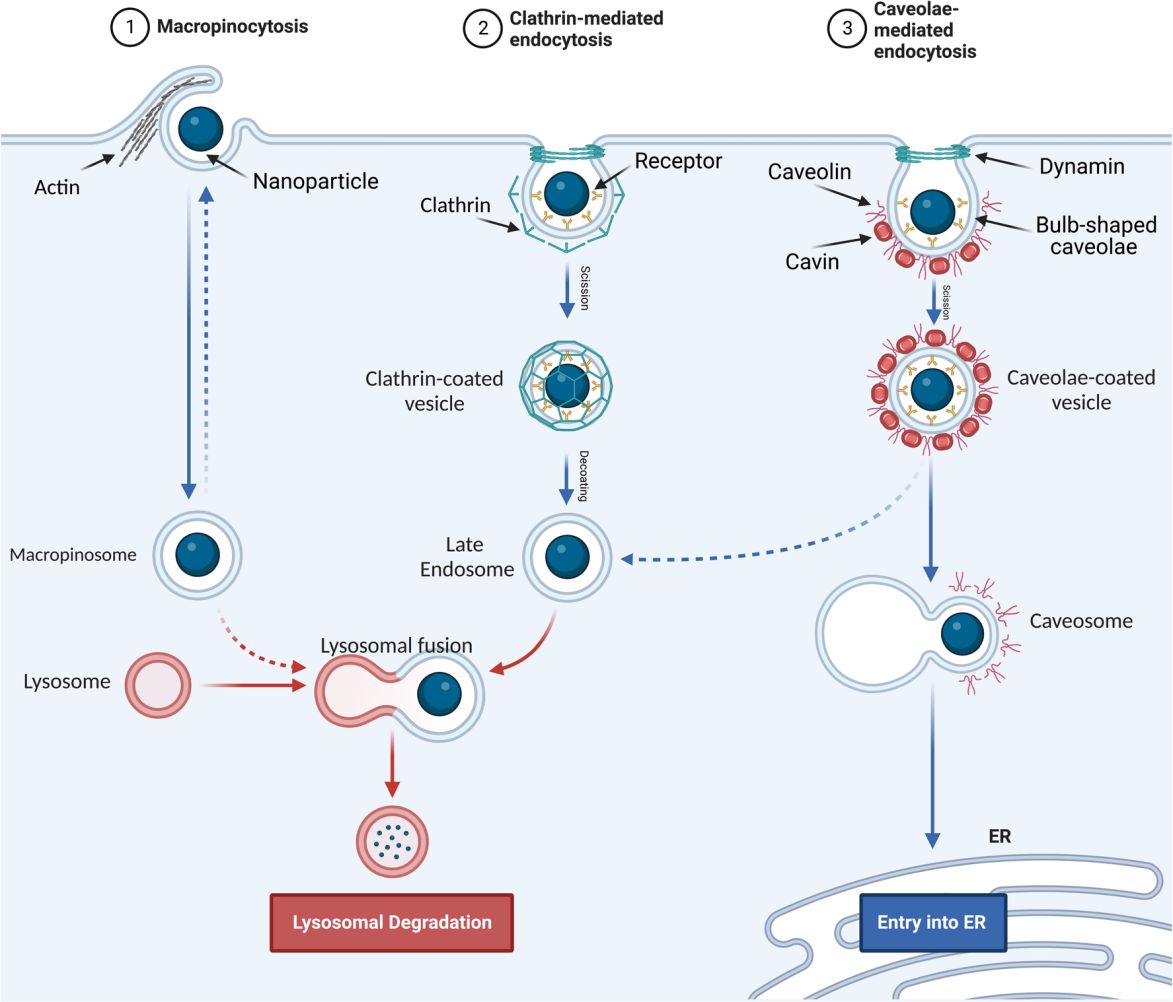
Pinocytosis internalization mechanisms. (1) Macropinocytosis. (2) Clathrin-mediated endocytosis. (3) Caveolae-mediated endocytosis.

This mechanism involves cytoskeleton rearrangements that induce the formation of membrane ruffles, which fold back, resulting in the formation of large intracellular vacuoles (0.1–5 μm)^51^ referred to as macropinosomes (see Fig. 2). Macropinocytosis is actin-dependent endocytosis, while it is independent of clathrin and membrane receptors.^52^

#### 2.2.2 Clathrin-mediated endocytosis

Clathrin-mediated endocytosis is the main process for the internalization of many NPs, which is used by all eukaryotic cells to internalize small particles and nutrients such as cholesterol. When the plasma membrane is rich in clathrin and ligand–receptor complexes start to form on the cell membrane surface, a cage of clathrin starts to form around the vesicle, resulting in vesicles with a diameter of 100–150 nm (see Fig. 2).^43,49,53^

#### 2.2.3 Caveolae-mediated endocytosis

Caveolae are bulb-shaped invaginations in the plasma membrane, which are 50–80 nm in size. These vesicles are coated by caveolin and cavin and detached from the membrane by dynamin, which is a 100 kDa GTPase (see Fig. 2).^43,54,55^

## 3. Role of physicochemical properties of NPs in cellular uptake and cytotoxicity

**Fig. 3:**
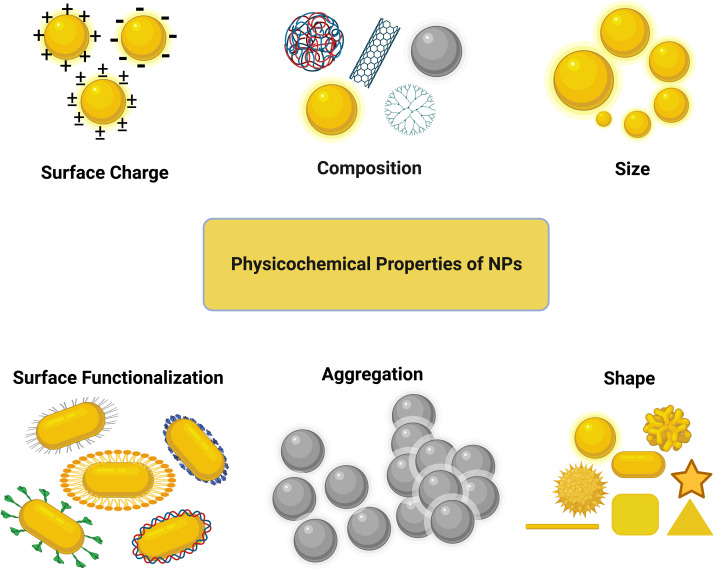
Physicochemical properties of NPs.

It is important to consider the physicochemical properties (size, shape, surface functionalization, surface chemistry, chemical composition, concentration, *etc.*) of NPs in their design for biomedical or other applications. The interactions of NPs with the cell membrane and organelles can significantly be altered at the bio-nano interface by these physicochemical properties, consequently changing the cellular uptake and nanotoxicity of the NPs. Therefore, before starting to assess the biological responses of NPs, thorough and proper characterization of the physicochemical properties of their core and surface should be performed.^56^ In this part, we mainly focus on the effect of the size, shape, and surface chemistry of NPs on their cytotoxicity and cellular uptake (see Fig. 3). The effect of the NP core composition is not discussed here given that the surface characteristics are more important than the bulk characteristics in this context.

### 3.1 Size

The size of NPs plays an important role in both their cellular uptake and cytotoxicity. Thus, it is considered a key factor when designing NPs for biomedical application. Due to the fact that NPs possess a size between atoms and bulk materials, they lie on the critical transition zone between two different worlds.^57,58^ It is worthy to mention that the original (primary) size of NPs differs from their hydrodynamic size in biological media.^59^ This is mainly because of the formation of a biomolecular corona and the aggregation of the NPs. In this case, the aggregation of NPs can be prevented by manipulating the balance of attractive and repulsive forces.^60^ For instance, Fe_3_O_4_ NPs can be stabilized with citrate, preventing their aggregation due to electrostatic repulsion.^61^ However, due to the formation of a biomolecular corona and the different ionic strengths of biological solutions compared to water, NPs may have new surface identity. Wei *et al.*^38^ performed a cytotoxicity study on the different sizes of TiO_2_ (5 and 200 nm) and Al_2_O_3_ (10 and 50 nm) NPs and observed the formation of aggregates in solution form when the NPs were suspended in cell medium without serum, where the sizes of all the NPs became 8–388-fold larger than their original sizes due to the higher ionic strength of the medium compared to water. Upon the addition of serum, the hydrodynamic sizes of the NPs decreased to only 1.6–10 folds larger than their original sizes. This is because the formation of the protein corona around the NPs prevented them from aggregating due to steric repulsion. The authors found that the smaller NPs (in terms of primary size, rather than hydrodynamic size) for both TiO_2_ and Al_2_O_3_ had higher cytotoxicity and much greater decrease in cell metabolic activity.

When studying the NP–cell membrane interaction mechanism dependence on the size of NPs, it was found that it has a strong influence. Specifically, large NPs (>60 nm) may cause steric hindrance, which prevents their interaction with the cell membrane.^62^ Conversely, NPs smaller than the cut-off size of receptor diffusion (<30 nm) may not recruit enough cell membrane receptors in the interaction region to overcome the elastic recoil force, preventing membrane wrapping from occurring.^63^ Moreover, the membrane receptors are known to form clusters that are 10–50 nm in size. Thus, a 50 nm NP, for example, needs to interact with only one receptor cluster, while a 500 nm NP must interact with several clusters simultaneously. This makes the internalization of the 50 nm NP energetically more favourable than the 500 nm NP.^44^

In general, smaller-sized NPs have been reported to have higher cellular uptake and higher cytotoxicity. For instance, Dong *et al.*^64^ reviewed 76 carefully chosen literature reports that included *in vitro* studies of the size-dependent cytotoxicity of amorphous silica NPs (aSiO_2_ NPs) and found that 76% of these papers showed that smaller-sized aSiO_2_ NPs exhibited greater cytotoxicity. However, it is important to consider that the cell type plays a role in this process given that it depends on the predominant pathway of cellular uptake in each different cell.^65,66^

For some NPs, the higher the cellular uptake of NPs, the greater their cytotoxicity.^67^ Nonetheless, there are some exceptions, where the cytotoxicity of NPs is independent of their cellular uptake. In these cases, the cytotoxicity is induced by sources other than amount of toxicant, including the NP high surface area, instability, and ion release. Gliga *et al.*^68^ found that 10 nm silver NPs (AgNPs) are more toxic to the human lung BEAS-2B cell line than other NPs with higher uptake ratios due to the release of more Ag^+^.

### 3.2 Shape

The shape of NPs can be controlled by manipulating the experimental conditions during their synthesis, such as supersaturation, reducing agents, temperature, surfactants, and secondary nucleation.^69^ There are many different shapes and geometries of NPs, such as spherical, rod, flower, star, disc, cubic, prismatic, and needle-like structures. The aspect ratio (AR), which is the proportion between width and height of NPs, is used to compare different shapes of NPs. For example, spherical AuNPs have an AR of 1, while Au nanorods (AuNRs) have a higher AR.

It was proven that the cellular uptake and cytotoxicity of NPs are affected by the AR of NPs. Given that AuNPs are common in many biomedical applications, many studies investigated their shape-dependent cellular uptake and cytotoxicity. For instance, Woźniak *et al.*^70^ compared the *in vitro* cytotoxicity profiles of different shapes and sizes of bare (non-coated) AuNPs in cancer (HeLa) and normal (HEK293T) cell lines. They found that Au nanospheres (AuNS) and AuNRs had higher cytotoxicity than star-, flower- and prism-shaped AuNPs. However, the sizes of these different AuNPs shapes also differed. Specifically, the AuNSs and AuNRs had smaller sizes (10 nm and 38 × 16 nm, respectively), while the flower-, prism-, and star-shaped AuNPs had larger sizes (∼370 nm, ∼160 nm, and ∼240 nm, respectively). Thus, their sizes may also play a crucial role in this cytotoxicity tendency, given that smaller NPs are known to have higher cellular uptake and aggregation rate inside the cell, which explains the observed cytotoxicity.

### 3.3 Surface charge

NPs can have negative, positive, or neutral surface charge depending on their surface functional groups.^71^ The surface charge can affect the NP–cell membrane interactions, protein corona, and consequently the cellular uptake of NPs.^72^ Therefore, it is one of the most important physicochemical properties to control when designing NPs for biomedical applications. Generally, reports have shown that charged NPs have higher cellular uptake than neutral NPs.^63^ The cell membrane is negatively charged due to the anionic head group of phospholipids and the existence of some carbohydrates, such as sialic acid.^73^ Considering this, cationic NPs, in most nonphagocytic cells, are taken up by the cells to a greater extent than anionic NPs. However, in some cases, anionic NPs have greater cellular uptake in phagocytic cells.^74,75^ The surface charge of NPs can also tune their cellular uptake pathway. For instance, Untener *et al.*^76^ reported that positively charged AuNRs had a higher extent of internalization compared to their negatively charged counterparts. It was found that cationic AuNRs were taken up through macropinocytosis and clathrin-mediated endocytosis, while anionic AuNRs were internalized through macropinocytosis and caveolae-related mechanisms.

**Fig. 4:**
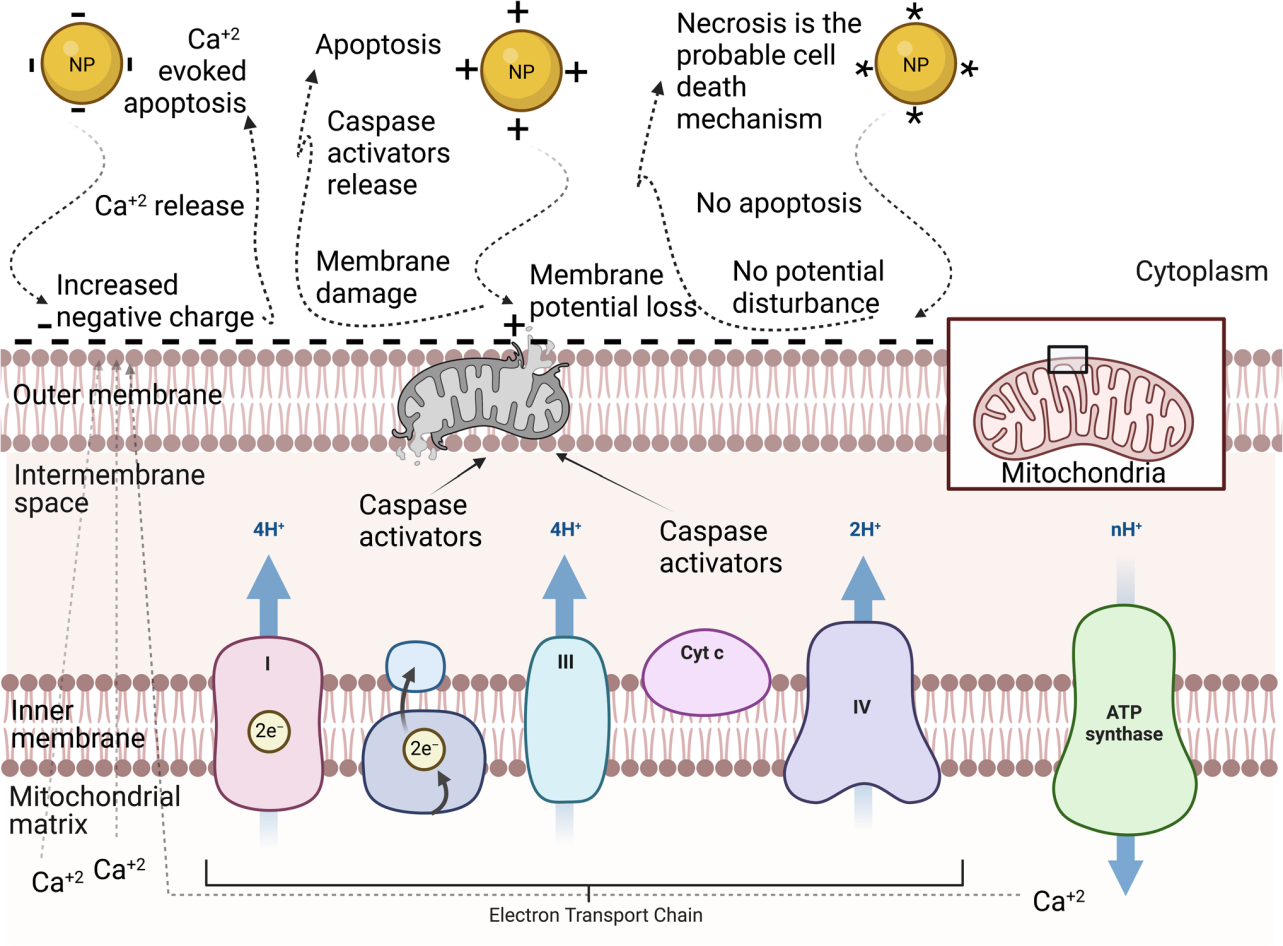
Schematic representation of the mitochondrial membrane: different NP surface charges induce different mechanisms of cell death.^77^ The neutrally charged NP (*) does not disrupt the mitochondrial membrane potential, and therefore apoptosis is not activated. The positively charged NP (+) disrupts the slight negative charge on the cytosolic side of the outer membrane, leading to a disruption in the mitochondrial membrane potential. The disruption damages the membrane and proteins, such as caspase activators, leak into the cytosol. The negatively charged NP (−) increases the negative charge on the outer membrane, which leads to a disruption in the mitochondrial membrane potential. The mitochondria compensate by releasing calcium ions that were stored in the matrix of the mitochondria. The spike in calcium induced apoptosis.^77^

The cytotoxicity of NPs is also, as expected, affected by their surface charge. Similar to the dependence of the cellular uptake of NPs on their surface charge, in nonphagocytic cells, charged NPs were found to be more cytotoxic than their neutral counterparts, with the positively charged NPs, in most cases, being more cytotoxic than negatively charged NPs.^74^ Moreover, the surface charge of NPs does not only affect their cytotoxicity level but also their mechanisms. A study by Schaeublin *et al.*^77^ showed that although both charged and neutral AuNPs were taken up in similar amounts and caused cell morphology disruption and decreased cell viability through ROS generation in a human keratinocyte cell line (HaCaT) model, only charged NPs caused significant mitochondrial stress. This suggested that the surface charge of AuNPs can affect the mechanism of cell death. Further investigations on mitochondrial-mediated toxicity revealed that neutral AuNPs did not affect the mitochondrial outer membrane potential, which has a slight negative charge, and thus apoptosis was not initiated, and the authors suggested that necrosis may be the cell death mechanism in this case. However, charged AuNPs affected this membrane in different ways. On the one hand, cationic AuNPs accumulated on the mitochondrial outer membrane due to its slight negative charge, which eventually damaged the membrane and caused the release of apoptotic proteins such as caspase-3 inducing mitochondrial-mediated apoptosis. On the other hand, anionic AuNPs increased this slight negative charge on the outer membrane, which forced the mitochondria, trying to adjust this potential disruption, to release the positively charged calcium ions into the cytosol, inducing calcium-evoked apoptosis (see Fig. 4).

### 3.4 Hydrophobicity

It has been shown that the hydrophobicity of NPs can affect the protein binding, cellular uptake, and cytotoxicity of NPs.^78–82^ The hydrophobicity and hydrophilicity of NPs can originate from the core or the functionalities of the NPs. In a recent systematic simulation study, Li *et al.*^78^ showed that changing the spikes of virus-like NPs (VLP) significantly altered the cellular uptake efficiency, while the effect of the core hydrophobicity of VLP was secondary. This study reported that VLP with hydrophobic or amphiphilic spikes were internalized more efficiently than that with hydrophilic spikes.

Generally, when keeping the other properties of NPs such as surface charge constant, their hydrophobicity has a positive trend with their cytotoxicity.^74^ Muthukumarasamyvel *et al.*^81^ controlled the hydrophobicity of dicationic amphiphile-stabilized AuNPs by conjugating the dicationic functionality with different numbers and locations of H and OH groups. The authors observed increasing anticancer or cytotoxicity properties with an increase in the surface hydrophobicity of the NPs against A549 lung cancer cells.

### 3.5 Surface functionalization

Changing the ligands on the surface of NPs will mostly tune the previous parameters (surface charge and hydrophobicity), which affects the protein corona, cellular uptake, and cytotoxicity of the NPs.^77,83,84^ However, the specific functionalities on the surface of NPs can be useful for targeting purposes. Here, overexpressed or unique receptors on the cell membrane are targeted by functionalizing the NPs with a complementary aptamer, protein, or antibody, which can specifically bind to the cell receptors. Tao *et al.*^85^ targeted cervical cancer cells through folic acid (FA)-poly(ethylene glycol)-*b*-poly(lactide-*co*-glycolide) blended NPs, which enhanced the efficacy of cancer chemotherapy through the targeted-delivery of anticancer drugs.

Lund *et al.*^86^ showed that AuNPs functionalized with 50% PEG–NH_2_/50% glucose had an eighteen-fold higher internalization rate than NPs functionalized with either PEG–NH_2_ or glucose alone due to their different organization patterns. Alternatively, Yeh *et al.*^87^ studied the role of ligand coordination of two quantum dots (QDs) on their cytotoxicity. The authors found that monothiol-functionalized QDs had greater levels of cytotoxicity compared to dithiol-functionalized QDs in HeLa cell lines. However, the monothiol-functionalized QDs had a higher charge density, and thus it is difficult to tell if this tendency is solely related to the ligand coordination or charge density.

Studying the dependency of cellular uptake and cytotoxicity on a certain physicochemical property of NPs can be very complex. For instance, changing their surface charge may lead to a change in hydrophobicity, hydrodynamic size, and protein corona. Furthermore, this may be done by changing the functionalities and coating of the NPs.^73–77^

Table 1 summarizes some recent studies exemplifying the effect of the physicochemical properties of NPs on their cellular uptake and cytotoxicity.

**Table 1:** Recent studies highlighting the influence of the physicochemical properties of NPs on their cellular uptake and cytotoxicity

Property	Parameter	NPs	Cell lines	Uptake mechanism	Cytotoxicity	Highlights	Ref.
Size	(5 and 200 nm)	TiO_2_	A549	—	ROS generation ↑	Smaller primary-sized NPs are more cytotoxic	38
	(10 and 50 nm)	Al_2_O_3_			Nutrient depletion		
Size	10, 40, 75 nm	Ag	BEAS-2B	Clathrin, caveolin/lipid raft, macropinocytosis and phagocytosis	Ag release (Trojan horse)	10 nm NPs are the most cytotoxic	68
					DNA damage		
Size	Spheres (15 nm-NP1, 45 nm-NP2, and 80 nm-NP3), rods (33 × 10 nm-NR), stars (15 nm-NS)	Au	SMCC-7721	Endocytosis (depends on corona)	Cell viability ↓	NS and NR are much more cytotoxic than the three spherical Au NPs. Cellular uptake in the order NP3 > NR > NP2 ≳ NP1 ∼ NS	88
Shape			GES-1				
Corona			4T1				
Size	Spheres (different sizes and coating), cubes, rods, prisms	Au	PC3	Endocytosis	Membrane damage	Increased uptake of smaller particles. AuNS are the most cytotoxic, followed by AuNPr, while both AuNR and AuNC are not toxic	89
Shape					Cell death		
Corona							
Size	Spheres (10 nm), flowers (370 nm), rods (41 nm), prisms (160 nm), stars (∼240 nm)	Au	HeLa	Endocytosis	Cell viability ↓	Au nanospheres and nanorods are more cytotoxic than star, flower and prism AuNPs	70
Shape			HEK293T				
Shape	Rods (*L* = 39 nm, *W* = 18 nm), stars (215 nm), spheres (6.3 nm)	Au	hFOB 1.19	Endocytosis & phagocytosis	Mitochondrial dysfunction	Au nanostars are the most cytotoxic to the three cell lines while AuNPs spheres are the least cytotoxic	90
			143B		Membrane damage, apoptosis		
			MG63				
			hTERT-HPNE				
Shape	Spherical and needle-like	PLGA–PEG	HepG2	Endocytosis	DNA damage, membrane damage, apoptosis	Spherical NPs have higher cellular uptake while needle-like NPs have greater cytotoxicity	91
			HeLa				
Shape	Rods	Al_2_O_3_	Rat ASTs	Phagocytosis	ROS generation, inflammatory response, metabolism changes, apoptosis	Nanorods have significantly greater cytotoxicity than nanoflakes against rat astrocytes	92
	Flakes						
Surface charge	Positive, negative, neutral	Au	HaCaT	Endocytosis	ROS generation, oxidative stress, mitochondrial stress, apoptosis, or necrosis	All three NPs generated significant ROS levels, but only charged NPs caused mitochondrial stress. Charged NPs caused cell death through apoptosis, while neutral NPs caused it through necrosis	77
Surface charge	Positive & negative with different zeta potentials	Polymeric	L929	—	Cell viability ↓	Cationic NPs are more cytotoxic that anionic NPS.	84
						As absolute zeta potential increases, cytotoxicity increases	
Surface charge	Positive/negative charge density and different hydrophobicity	Au	A549	Endocytosis	ROS generation	Positive trend in the cytotoxicity of NPs over their surface hydrophobicity	82
Hydrophobicity			HEK293		Apoptosis		
Hydrophobicity	Three dicationic amphiphile-stabilized	Au	A549	—	ROS generation	Positive trend in the cytotoxicity of NPs over their surface hydrophobicity	81
	AuNPs				Apoptosis		

## 4. Cytotoxicity assessment

**Fig. 5:**
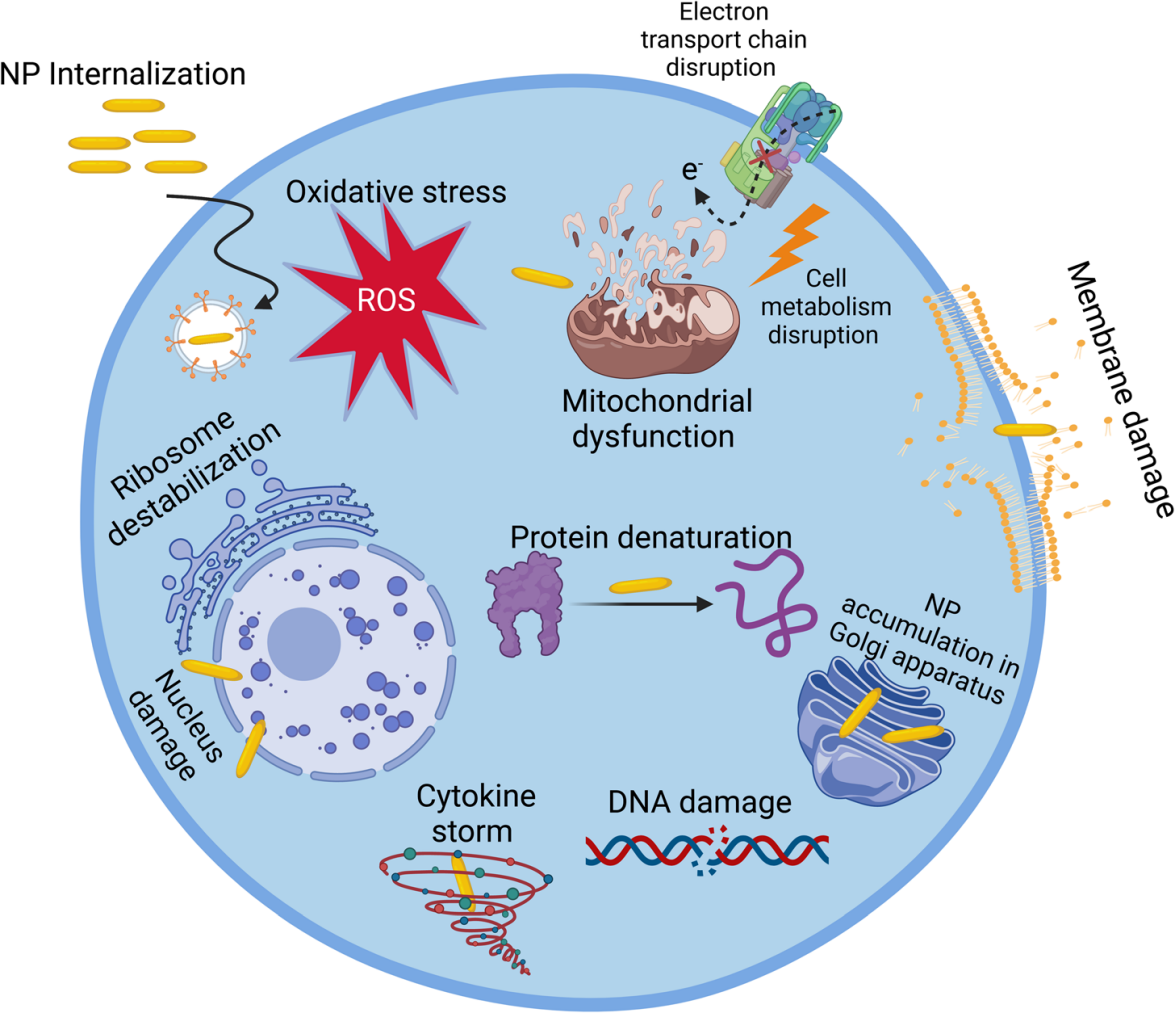
Various modes of action of NPs on cells.

*In vitro* cytotoxicity of NPs is assessed using cell models. Although this assessment does not replace the *in vivo* evaluation of their cytotoxicity, it represents a screening bridge between the investigation of the quality and *in vivo* application of materials.^56,93^ Herein, we focus on the *in vitro* assessment of nanotoxicity. In the case of *in vivo* assessment, readers are encouraged to read the wholistic review by Kumar *et al.*^94^ Many *in vitro* assays are used to investigate or measure the cytotoxicity of NPs. These assays can be categorized to five main categories including cell viability and proliferation, ROS generation, cell stress, cell morphology phenotyping, and cell–NP uptake assays.^56^Fig. 5 demonstrates some pathways of the effect of NPs on cells.

### 4.1 Cell viability and proliferation

Cell viability assays focus on investigating the cell metabolic activity and mitochondrial enzymes, such as lactate dehydrogenase (LDH), an enzyme that regulates pyruvate and lactate levels through nicotinamide adenine dinucleotide (NAD) oxidation.^93,95^ Tetrazolium salts can react with the mitochondrial dehydrogenase enzymes. This reaction leads to the cleavage of the tetrazolium ring and conversion of these salts into a colored formazan form, which can be detected using colorimetry-spectroscopy. The detected activity of these enzymes is an indication of the cell viability. One of the most commonly used tetrazolium salts for assessing the cytotoxicity of NPs is the 3-(4,5-dimethylthiazol-2-yl)-2,5-diphenyltetrazolium bromide (MTT) assay.^96^ The other tetrazolium salts used include 3-(4,5-dimethylthiazol-2-yl)-5-(3-carboxymethoxyphenyl)-2-(4-sulfo phenyl)-2*H*-tetrazolium (MTS), iodonitrotetrazolium (INT), and 4-[3-(4-iodophenyl)-2-(4-nitrophenyl)-2*H*-5-tetrazolio]-1,3-benzene disulfonate (WST-1), which different to the previous salts, produce the water-soluble formazan. Other colorimetric/fluorimetric cytotoxicity assays are also used, for example, neutral red, trypan blue, lactate dehydrogenase (LDH), mitochondrial membrane potential (MMP), and Alamar Blue (resazurin) assays.

**Fig. 6:**
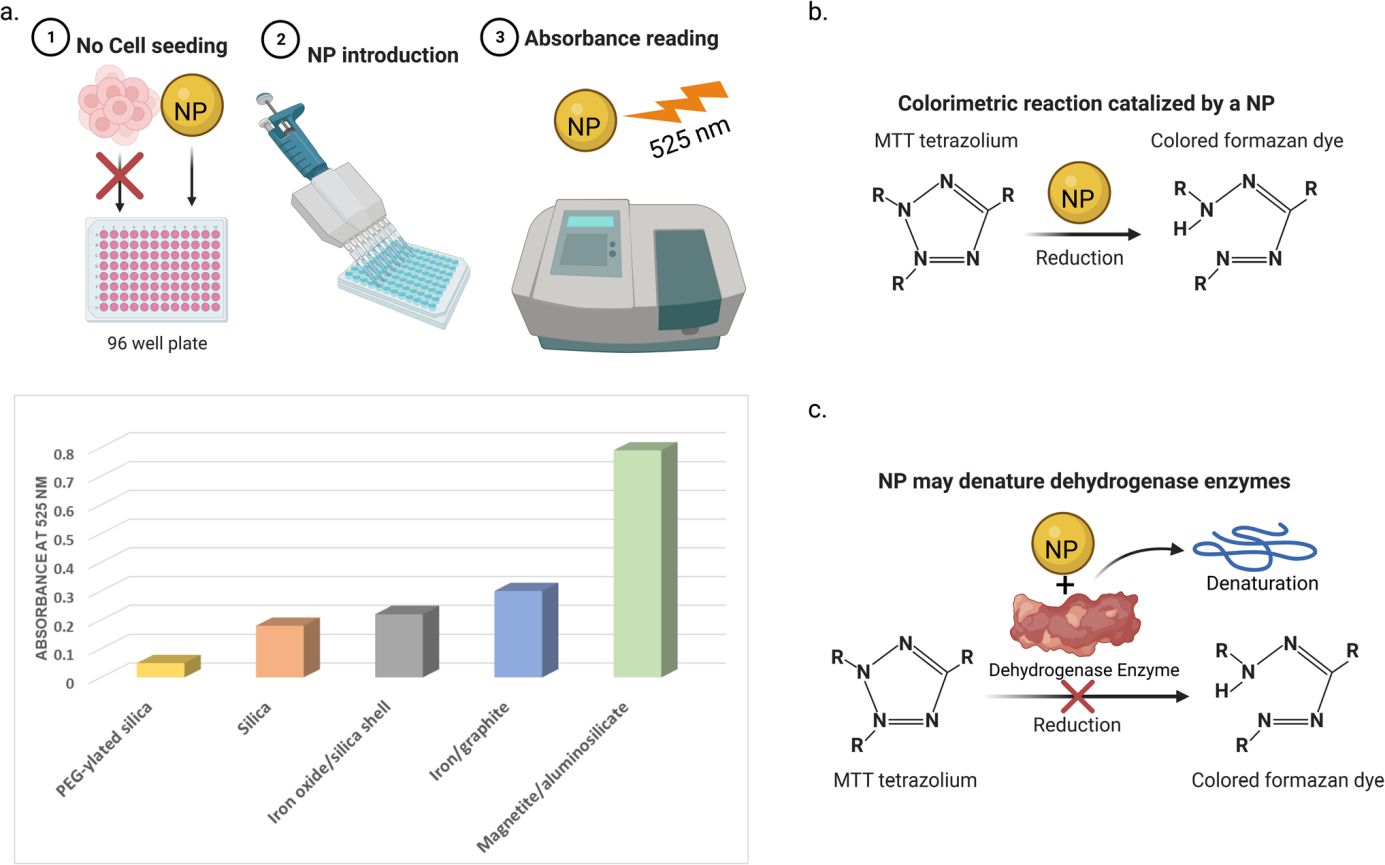
Three ways of NPs interference with MTT cell viability test. (a) NPs in the absence of cells showed light absorbance at the same wavelength of MTT assay (525 nm in this study). The absorbance of the 5 NPs was measured at a concentration of 32 μg mL^−1^. Data was obtained from ref. 100 (b) NPs can catalyze the reduction of the MTT (or another test agent) to its colored (or fluorescent) form without the existence of cell enzymes. (c) NPs may adsorb and denature the cell enzymes that reduce the MTT dye to its colored form, giving false results.

Many types of interference between NPs and cell viability assays have been reported. One way is the adsorption of the mitochondrial activity-related proteins on the NP surfaces. This may lead to the enzyme denaturation, giving false results of the cell viability profiles.^97^ For instance, Stueker *et al.*^98^ used molecular dynamics simulation to investigate the effect of LDH enzyme binding on functionalized AuNPs. The authors observed that the dynamics of the side chains of the enzyme were largely constrained in all four active sites. Another way of interference is that the light absorbance spectra of the NPs can interfere with the absorption window of the assay, leading to false colorimetric measurements.^99^ For example, Díaz *et al.*^100^ reported that five NPs (magnetic iron/graphite, magnetite/silica, bare and poly(ethylene glycol)(PEG)–ylated silica, and magnetite/FAU zeolite) in culture medium after 72 h (in the absence of cells) showed absorbance at the same wavelength (525 nm) used in the MTT assay. This absorbance increases with the NP concentration, depending on their type. The third way of interference is that NPs may interact with the assay reagents. For instance, Hoshino *et al.*^101^ reported that cysteamine-coated quantum dots catalytically reduced MTT to formazan without cellular metabolism taking place (see Fig. 6).

### 4.2 ROS generation and oxidative stress

Reactive oxygen species (ROS) are a type of unstable molecule (free radicals) that contain oxygen and can easily react with the other molecules in cells. The ROS include the superoxide anion (O_2_˙^−^), hydrogen peroxide (H_2_O_2_), and hydroxyl radical (HO˙). ROS are normally produced by cells at certain levels to maintain regular metabolism and homeostasis, which are considered as critical signalling molecules in cell proliferation and survival.^99,102^ However, they may be produced through interactions with exogenous sources such as NPs. If this event produces excessive ROS that the cellular antioxidant defense system (enzymatic antioxidants such as glutathione (GSH) peroxidases) cannot handle, oxidative stress is triggered.^102,103^ This may lead to the destruction of organelles and bio-molecules, including triggering membrane damage, lipid peroxidation, DNA damage, protein damage, apoptosis, necrosis, and inflammatory response, leading to many diseases such as cancer, diabetes, neurodegenerative, and cornea diseases.^103,104^

NPs can generate ROS by acting as a catalyst in ROS generation reactions. For instance, Higashi *et al.*^105^ reported the catalytic generation of ROS by AuNPs and showed that this reaction can be controlled by changing conditions such as the type, concentration, and pH of the NP solution.

ROS detection can be performed by the direct measurement of ROS levels or the measurement of their oxidative damage or other outcomes.^106^ Some direct methods for the detection of ROS are fluorescein-compound-based tests and electron paramagnetic resonance (EPR). The reactive fluorescein probes 2′,7′-difluorescein-diacetate (DCFH-DA) and dichlorodihydro-fluorescein diacetate (H_2_ DCFDA) are non-fluorescent; however, when they are exposed to the cell cytosol enzymes, they get hydrolysed. Then, the cellular ROS oxidize them into a highly fluorescent compound, dichlorofluorescein (DCF), yielding an optical ROS concentration-dependent response, which can be measured using fluorescence microscopy or flow cytometry.^107^ Alternatively, indirect approaches for the detection of ROS include many assays that depend on the stimulated oxidative effect of the ROS. One approach is by measuring the enzymatic or non-enzymatic antioxidants levels.^106^ Oxidative stress can also be assessed by measuring the oxidative damage of the cell biomolecules. These damaged biomolecules include proteins, lipids, and DNA and can be detected by measuring the protein carbonyl content,^108,109^ malondialdehyde levels,^110,111^ and 8-oxo-2′-deoxyguanosine (8-OdG) lesion,^112–114^ respectively. Other genotoxicity assays include the comet, Ames, micronucleus, and chromosome aberration assays.^115^

During the course of measuring NP-induced ROS generation and oxidative stress, NP-assay interferences may occur.^116,117^ In colorimetric- and fluorimetric-dependent assays, NPs may interact with the final form of the dyes in a way that alters, by enhancing or reducing, the absorbance or fluorescence of the dye. For example, Aranda *et al.*^116^ observed the quenching effect of several NPs on the dye fluorescence emission in the DCFH-DA assay, which was correlated with the cellular uptake of the NPs. The authors suggested a threshold concentration of NPs at which their oxidative effect can be detected, and they proposed that changing the experimental conditions can reduce this interference. Conversely, Pfaller *et al.*^117^ reported the dye fluorescence enhancement of the DCFH-DA assay in the presence of Au or Fe_2_O_3_ NPs. This confirms that both scenarios (quenching and enhancement) may occur due to NP–probe interactions during colorimetric- and fluorimetric assays.

### 4.3 Inflammatory response

The inflammatory response induced by NPs in a cell line can be measured by detecting the produced inflammatory biomarkers. Macrophages and other cells release many cytokines, which play a crucial role in cell communication in the immune system by, for instance, promoting inflammation. Interleukins (ILs), such as IL-1β, IL-6, IL-8, and IL-10, in addition to other cytokines, such as tumor necrosis factor TNF-α and granulocyte-macrophage colony-stimulating factor (GM-CSF), play a central role in inflammation regulation. The expression of these biomarkers can be assayed to determine the inflammatory response caused by NPs. ELISA (enzyme-linked immunosorbent assay) or western blotting, and electrophoretic mobility shift assays (EMSAs) or real-time polymerase chain reaction (RT-PCR) systems are used for the measurement of cytokines and the related genetic expressions, respectively.^118–123^

**Fig. 7:**
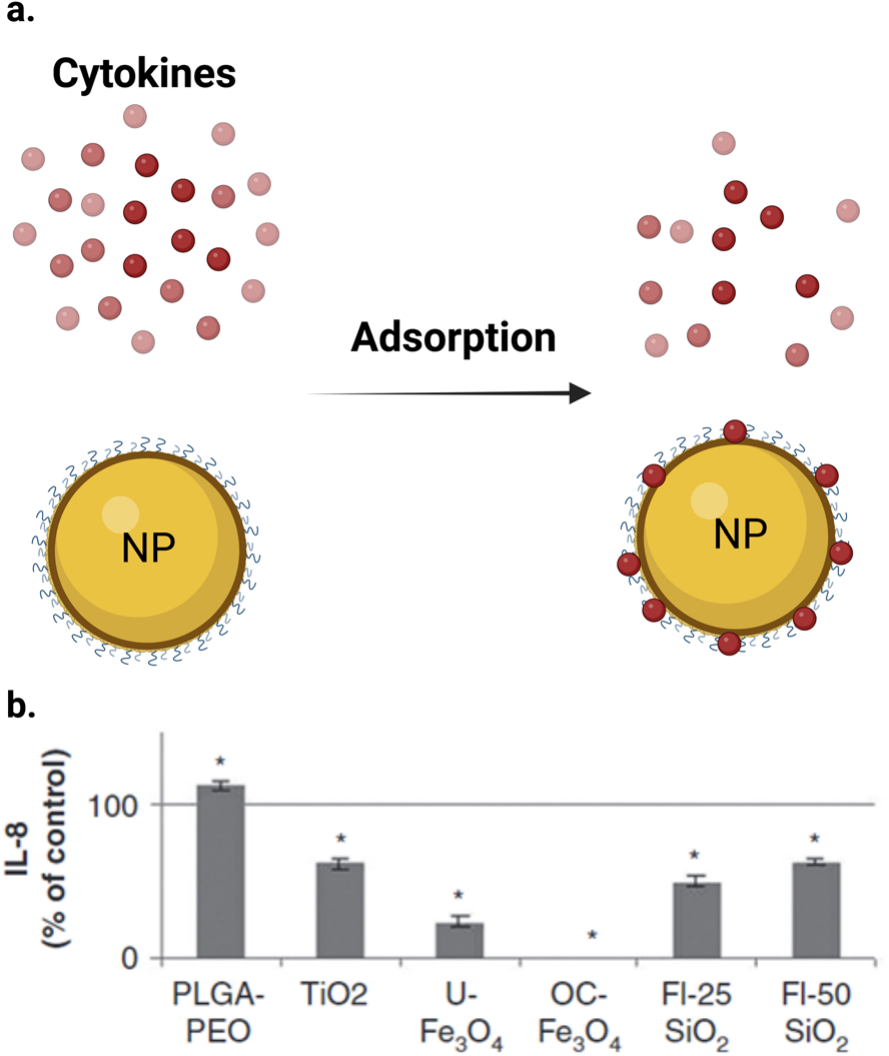
Interference of NPs with ELISA assay. (a) NPs can adsorb different cytokines on their surface. (b) IL-8 were quantified by ELISA after 24 h of incubation with NPs at 75 μg cm^−2^ after elimination of particles by centrifugation. Results (*n* = 6) are expressed as % of control (cytokine incubated in the absence of NPs). *Significantly different from the control (*p* < 0.05 ANOVA followed by Dunnett's test). (b) Is reproduced from ref. 122 with permission from Taylor & Francis. Copyright 2015.

NPs were reported to induce an inflammatory response in different cell lines. Many studies used conventional assays to measure this response.^119–121^ However, these assays can also interfere with NPs during the measurement of inflammatory response in cell lines. Some inflammatory cytokines were reported to be adsorbed on the NP surface, causing interference (Fig. 7a). Guadagnini *et al.*^122^ investigated the interferences of different NPs with some *in vitro* cytotoxicity assays. The authors reported that Fe_2_O_3_, TiO_2_, and SiO_2_ NPs significantly adsorbed IL-6, IL-8, and GM-CSF cytokines on their surfaces at different levels at a NP concentration of 75 μg cm^−2^. Fig. 7b shows that all the studied NPs adsorbed the IL-8 cytokine except PLGA–PEO NPs, which surprisingly increased the apparent level of cytokines, probably due to the stabilization of the peptides and their protection from proteolysis. In the case of other NPs, the level of adsorption depends on the NPs and the cytokine studied. OC–Fe_3_O_4_ NPs are the most cytokine-adsorbing NPs tested given that cytokines could not be detected in the supernatants. Furthermore, Piret *et al.*^123^ observed a high inter-laboratory variability for the ELISA assay for IL1-β and TNF-α measurements and they suggested that testing of NP-cytotoxicity assay interferences should be always performed. Readers should kindly refer to ref. 122 for more information about the interference between different assay and NPs and some solutions to this problem.

### 4.4 Apoptosis and necrosis

Apoptosis is a programmed cell death pattern,^124^ while necrosis is an unprogrammed cell death.^125^ Both patterns of cell death can be an outcome of NP treatment.^126–131^ Nickel ferrite (NiFe_2_O_4_),^126^ TiO_2_,^127^ Fe_2_O_3_,^128^ hydroxyapatite,^129^ and Ag^130^ NPs induced apoptosis in A549, BEAS-2B, ECV304, C6, and HepG2 cell lines, respectively. Alternatively, Reus *et al.*^131^ reported dose-dependent cell necrosis induced by SiO_2_ NPs in BALB/c 3T3 cell line. Apoptotic cell death is mostly non-inflammatory, while necrotic cell death can be inflammatory.^132^ Both pathways are extremes, and many cases are a complex combination of both. For instance, Kumar *et al.*^133^ observed that AgNPs caused cell death in L-929 fibroblast cell lines in association with both necrosis and apoptosis. The cell death pathway is controlled by many parameters such as the surface charge, concentration, and exposure time of NPs. Schaeublin *et al.*^77^ reported that charged AuNPs caused cell death through apoptosis, while neutral AuNPs caused it through necrosis (see Fig. 4).

**Fig. 8:**
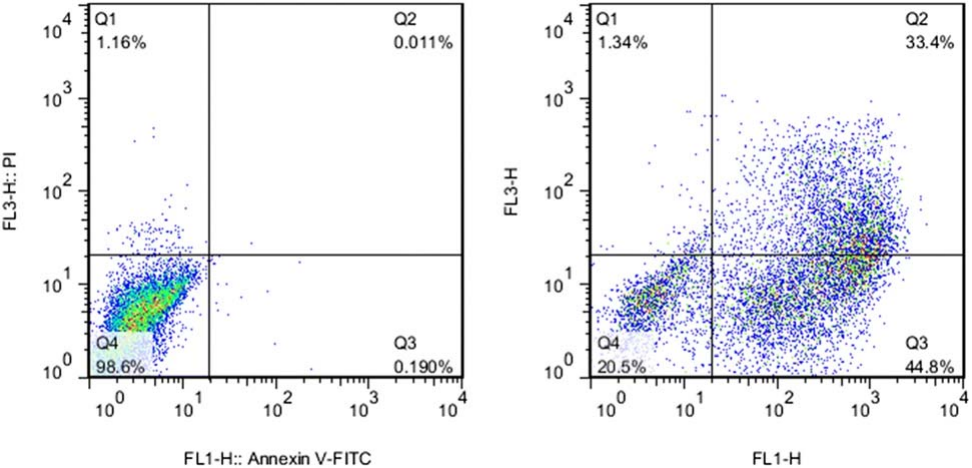
Evaluation of apoptosis and necrosis activities in MCF-7 cells using Annexin-V/PI staining. (Left) Untreated cells (control) and (Right) cells treated with ZnPNPs. Reproduced from ref. 135, with permission. Copyright © 2020, Springer Science Business Media, LLC, part of Springer Nature.

Many assays are used to detect apoptosis and necrosis. Phosphatidylserine (PS) migration to the extracellular side of the cell membrane and caspase activation into initiator and effector enzymes are two events that accompany apoptosis and can be used as markers to detect it. Externalized PS on the surface of the cell can be detected using fluorescein isothiocyanate (FITC)-labelled Annexin-V. Annexin-V specifically binds to the exposed PS on the cell surface in the early apoptotic cells, and then can be measured *via* flow cytometry or fluorescent microscopy. Alternatively, the membrane-impermeable propidium iodide (PI) dye exclusion assay is used for the identification of cellular necrosis. PI binds to DNA in the nucleus and stains it only when the cell membrane integrity is lost (which is an event that accompanies necrosis). Thus, a combination of the above-mentioned assays can determine the pattern of cell death.^134^ For instance, Vafaei *et al.*^135^ used the Annexin V-FITC/PI staining kit to study the apoptotic efficacy of zinc-phosphate NPs (ZnPNPs) against the MCF-7 breast cancer cell line. The untreated cells with NPs showed a live cell (Annexin V-FITC−/PI−) percentage of 98.6%. Conversely, after exposure to ZnPNPs, the apoptotic cell (Annexin V-FITC+/PI−) ratio increased from 0.190% to 44.8% and the necrotic cell (Annexin V-FITC+/PI+) percentage increased to 1.34% (see Fig. 8).

Flow cytometry-based assays have negligible NP interferences.^133^ Bancos *et al.*^136^ reported that SiO_2_ NPs have low or no interference with flow cytometry assays. However, other colorimetric and fluorimetric-based assays face the same problems mentioned in the previous sections.

## 5. Metabolomics for the cytotoxicity assessment of NPs

In general, most studies on the cytotoxicity of NPs use the conventional (phenotypic) assays. However, many of these assays, as mentioned before, have been reported to interfere with the NPs because of their color, fluorescence, chemical activity, light scattering, *etc.* Thus, to precisely reveal the cytotoxicity of NPs, it is necessary to use a combination of more than two assays. This involves testing for NP interferences and eliminating them by changing experimental conditions or comparing the results of two similar tests, which is a complex and time-consuming process. However, many reports only used one or two cytotoxicity assays and ignored any potential interference with NPs.^137^ In addition, even though the conventional cytotoxicity assays can reveal that a certain cytotoxicity outcome happened, these assays are limited in terms of detecting the molecular information that caused this event.

The current toxicological assays need to be updated and new tools should be incorporated progressively in this field.^138^ A more advanced and emerging approach to study the toxicity of particles is the “omics” technique, which is based on the change in epigenome, transcriptome, proteome, genome, lipidome, and metabolome profiles introduced by internal or external stimuli. In increasing number of studies are using this approach to investigate the *in vitro* and *in vivo* toxicity induced by NPs. The determination of new targets and biomarkers for NP toxicity is one of the strengths of the omics technique. Moreover, the omics technique has high sensitivity, which is useful because of the low levels of environmental exposure to NPs that sometimes cannot be detected using the conventional assays.^39^ Another strength is that unlike the conventional assays, the omics technique has low or no interferences with NPs.^39,122^

In the field of toxicology, the most related omics discipline is metabolomics.^139^ Metabolomics, one of the newest in the omics era, is an emerging field, which is broadly defined as the comprehensive measurement of all metabolites and low-molecular-weight molecules in a biological specimen (tissues, cells, fluids, or organisms),^40^ and is one of the most powerful bioanalytical strategies that allow a picture of the changes of metabolites levels of an organism to be obtained during the course of a biological process either as a footprint (analysis of extracellular metabolites) or fingerprint (analysis of intracellular metabolites).^41^ The detailed analysis of low molecular weight compounds provided by nuclear magnetic resonance (NMR) spectroscopy or mass spectrometry (MS), besides the analysis performed by the powerful chemometric software (MetaboAnalyst),^140^ provides an accurate and quick detection and comparison of many types of chemical entities including carbohydrates, amino acids, nucleotides, lipids, steroids, fatty acids, and their derivatives, which are produced by cell metabolism.^141^

**Fig. 9:**
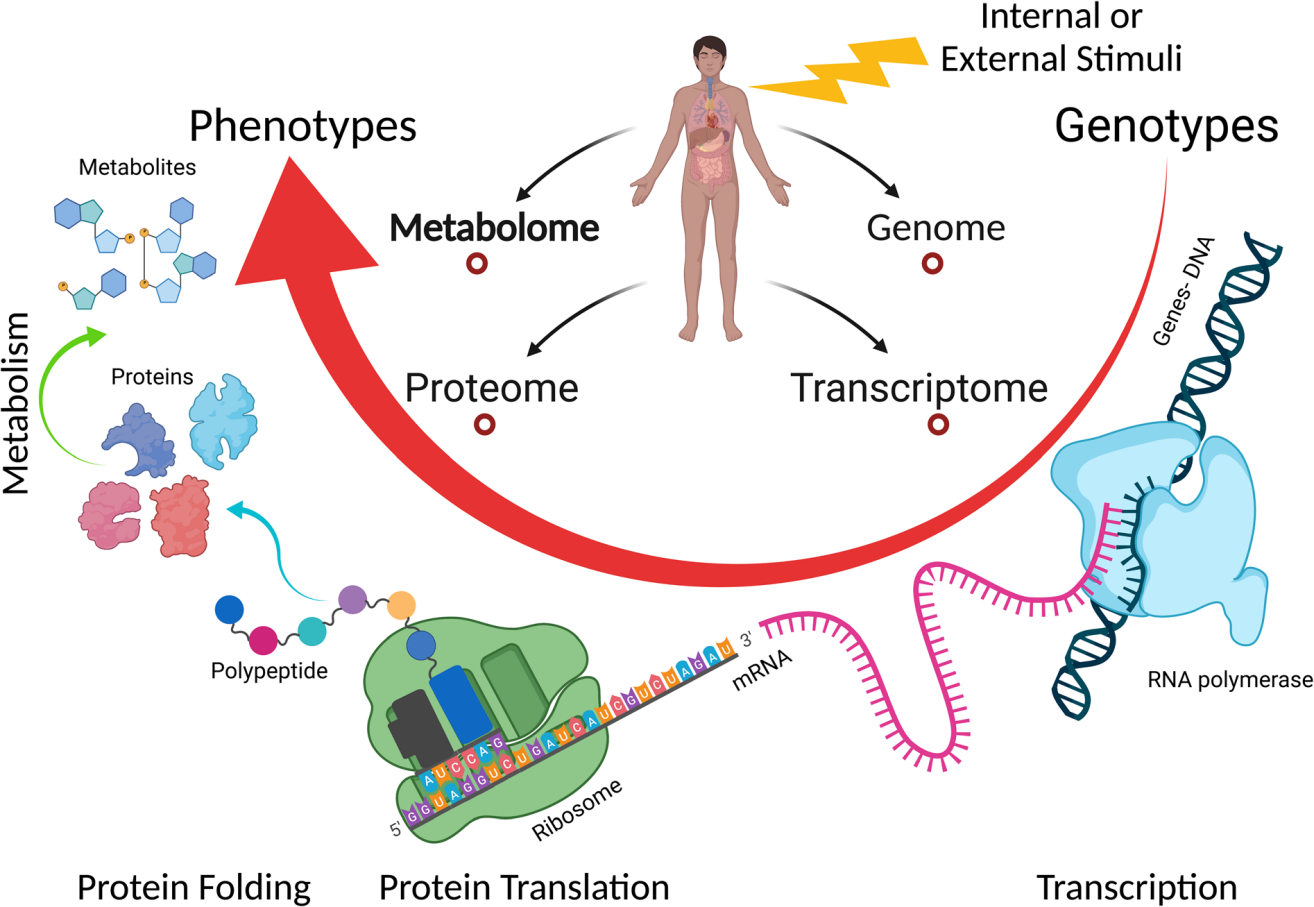
Overview of the connection of the main omics-sciences: genomics, transcriptomics, proteomics, and metabolomics. Metabolomics represents the final output of cellular processes.

Currently, metabolomics is applied in many fields such as disease fingerprinting, biotechnology, environmental and plant research, toxicology and safety research, clinical medicine, and pharmacology.^139,142,143^ Our group has been investigating the metabolic changes in serum, urine, and feces induced by different diseases such as lung cancer and diabetes, or other stimuli such as kidney transplant.^144–146^ Due to the non-invasive sampling in the metabolomic approach, the relatively low number of metabolites (compared to transcripts and proteins), and good level of knowledge about the role of most metabolites, metabolomics provides a well-grounded and precise methodology to investigate the biochemical effects and toxicity of NPs,^139,147^ and it can present insight into the genotype and phenotype changes with a biological response.^148^ Moreover, single-cell metabolomics is achievable today, making it possible to determine phenotypic heterogeneity among individual cells.^149^ Many cellular activities such as intercellular signal transduction, energy transfer, cell proliferation, and differentiation occur at the metabolite molecular level and are regulated by the presence and level of specific metabolites. Furthermore, metabolites are the end result of the expression of functional genome, transcriptome, and proteome (see Fig. 9).^150,151^ This indicates that metabolomics can detect many NP cytotoxicity outcomes and reveal the molecular information behind these events even at low levels of NP exposure and with no interferences. Therefore, it is a great tool in nanotoxicology, which is being applied to reveal the effect and toxicity of NPs in many fields including environmental and agricultural fields^152–154^ and cancer research.^155^ Metabolomics can help in better understanding of the transition from *in vitro* to *in vivo* systems of NP toxicity and its effect given that it is applied in both types of experiment.^156,157^ Furthermore, metabolomics can be combined with other omics techniques to provide a more comprehensive understanding of the effects of NPs on cells.^158–160^

When comparing NMR- and MS-based metabolomics, generally, NMR has lower sensitivity than MS, and thus it is considered more suitable to analyze extracellular metabolites (exometabolome), which is done by the analysis of the cell culture media. Alternatively, the more sensitive MS techniques are more suitable for the analysis of relatively low levels of intracellular metabolites (endometabolome), especially when isolated from a limited number of cells. However, both analysis techniques are complementary and should be used simultaneously to maximize the metabolic window.

**Fig. 10:**
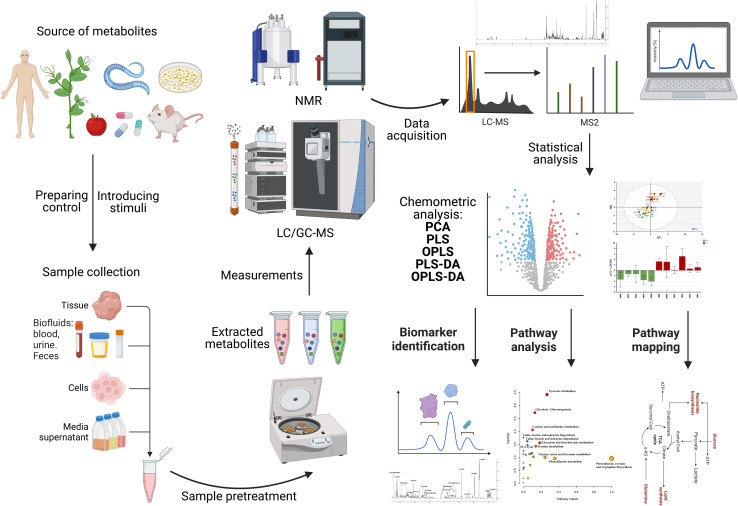
Metabolomics workflow for NMR- or MS-based metabolomics. DA: discriminant analysis; PCA: principal component analysis; PLS: partial least squares; and OPLS: orthogonal partial least squares.

This emerging technique has not yet been widely applied for the investigation of NP cytotoxicity in *in vitro* systems and more research needs to be done on different NPs and cell lines. In this section, we focus on the metabolic changes induced by different NPs in different cells *in vitro*. The workflow of a metabolomics experiment is demonstrated in Fig. 10. This review does not go into detail on the workflow of metabolomics. In this case, for a detailed demonstration of how metabolomic workflows generate data, the reader is directed to read the following reviews and book chapters.^39,160–166^

### 5.1 AuNPs

Gold nanoparticles (AuNPs) are very common in the biomedical field. AuNPs have many unique properties such as ease of synthesis, tunable size, ease of surface modification, surface plasmon resonance (SPR), and X-ray attenuation.^167^ This makes them the center of attention in many applications, including the growing field of nanomedicine, biosensors, targeted drug delivery, radiation therapy, photothermal therapy, biomedical imaging, and cancer diagnostics and therapeutics.^155^

**Fig. 11:**
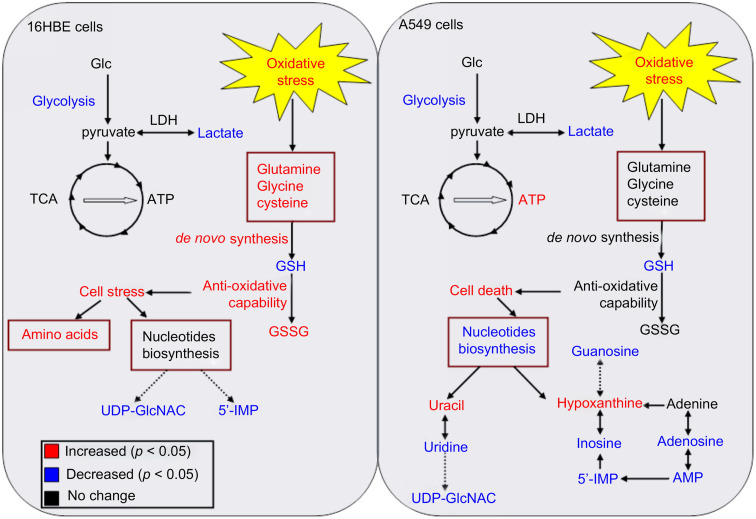
Summary of various metabolic responses of A549 (Right) and 16HBE (Left) cells to AuNR exposure. Metabolites in red or blue represent a significant increase or decrease in their levels, respectively, in the AuNR-treated groups compared with the non-treated groups. (This figure has been reprinted from ref. 169 with permission. Copyright © 2013, Elsevier Ltd).

Metabolomics is used in several studies to assess the cytotoxicity of AuNPs and reveal their molecular information. Au nanorods (AuNRs) are one example of AuNPs that have strong absorption in the near-infrared spectral region and can be used in tumor thermal therapy (hyperthermia), and also in targeted tumor therapy. Wang *et al.*^168^ observed, using conventional assays, that AuNRs have a unique influence on cell viability by causing the death of cancer cells (A549 cell line), while having negligible effect on normal cells (16HBE and MSC cell lines). The authors showed that AuNRs were released from the lysosome of cancer cells, and then translocated into the mitochondria, causing oxidative stress by the production of ROS. Alternatively, the normal cells had more intact lysosomes, and thus the AuNRs were not released in the cell cytoplasm. However, the molecular information during this cellular translocation was unclear. Later, the same group,^169^ used a metabonomic approach, a subset of metabolomics,^170^ by applying ^1^H NMR and multivariate data analysis, to study the metabolic change with time during the exposure of A595 and 16HBE cell lines to AuNRs. The authors found that both cell lines had intracellular disruption by the reduction of lactate levels and by causing oxidative stress. However, the normal cells resisted this oxidative stress by *de novo* GSH synthesis, unlike the cancer cells, which did not trigger this pathway, causing severe damage of their mitochondria (see Fig. 11). The metabonomic study further indicated the downregulation of nucleosides and nucleotides in the cancer cells, indicating cell death. Alternatively, the amino acid levels were upregulated in the normal cells, indicating cell stress. This study shows the usefulness of metabolomics in revealing the molecular information of the effect of NPs on cells, after conventional assays played the role of a general scanner for these effects.

Metabolomics can help in identifying biomarkers for NP cytotoxicity. For example, Xu *et al.*^171^ investigated the potential harmful effects of AuNRs on male reproduction by studying the metabolic change in spermatocyte-derived cells (GC-2) and Sertoli (TM-4) cell line after exposure to 10 nM of AuNRs. Employing metabolomics, the authors observed a strong downregulation in glycine levels in TM-4 cells, while there was no significant change in GC-2 cells. To identify what may accompany this reduction of glycine (potential biomarker), high content screening (HCS) and JC staining were used, and it was found that AuNRs decreased the membrane permeability and mitochondrial membrane potential of TM-4 cells. Moreover, the authors observed a disruption in the mRNA and protein levels of blood–testis barrier (BTB) factors using RT-PCR and western blot. Then, to confirm that glycine is a biomarker for these events, the authors repeated the experiments after adding glycine to the medium and noticed that the cells recovered from the previous harmful effects. This experiment reveals that glycine can be recognized as a biomarker to the changes in membrane permeability, mitochondrial membrane potential, and blood–testis barrier (BTB) factors in further similar experiments.

Huang *et al.*^172^ observed that spherical AuNPs (20 nm) were not cytotoxic against the human dermal fibroblast (HDF) cell line. The authors combined bioinformatics with metabolomics to determine the molecular information of this toxicity resistance. Firstly, they detected that 29, 30 and 27 metabolites were differentially expressed in HDFs after 4, 8, and 24 h treatment with AuNPs, respectively. Among them, only six metabolites were determined to be key metabolites using bioinformatics techniques including expression pattern analysis and metabolic pathway analysis using MetaboAnalyst online tool. The key metabolic pathway was identified to be the GSH pathway with GSH as the key metabolite. Subsequently, these results were verified and it was found that the increase in GSH levels after AuNP treatment may be the reason behind the toxicity resistance behaviour of the cells, given that GSH can trigger an oxidative stress protection mechanism that helps in avoiding cytotoxicity.^169^ This reveals that GSH can be considered as a biomarker for oxidative stress resistance.

Lindeque *et al.*^173^ used MS metabolomics to study the effect of citrate-, poly-(sodium styrene sulfonate)-, and poly-vinylpyrrolidone (PVP)-capped AuNPs on the intracellular metabolites of HepG2 cells. Surprisingly, after 3 h of treatment, a holistic depletion of intracellular metabolites was observed for all the capped AuNPs. Usually, metabolic changes result in the upregulation of the metabolite levels because of secondary pathways, clearance issues, and reduced enzyme functionality.^174^ Firstly, the authors suggested that a loss of cell membrane integrity happened, but the exometabolomic data, measured using the NMR technique, was not consistent with this reasoning. Subsequently, they hypothesized that the AuNPs bind to the intracellular metabolites with or without replacing the surface coatings.

Gioria *et al.*^175^ combined proteomics and metabolomics to gain a further understanding of the effects of two sizes, *i.e.*, 5 and 30 nm, of AuNPs on the human colon adenocarcinoma Caco-2 cell line. The proteome and metabolome are directly interconnected and influence each other given that the protein levels can change the metabolic profile of a cell system and *vice versa*. Genomics and transcriptomics were excluded from this study due to their restricted value given that they provide limited information about phenotyping. The authors used liquid chromatography high-resolution tandem mass spectrometry (LC-HRMS/MS) and two-dimensional gel electrophoresis (2DE) to obtain qualitative and quantitative data of de-regulated metabolites and proteins, respectively. Subsequently, the data was combined and interpreted using systems biology analysis. After 72 h of exposure to AuNPs, 61 proteins and 35 metabolites in the cell extract were identified to be up-/down-regulated. The internalization mechanism was found to be endocytosis due to the downregulation of the SH3GL1 and EAA1 proteins, which are involved in the endocytic pathway. The smaller-sized AuNPs caused a greater number of de-regulated proteins and metabolites due to their higher internalization in the cells. Concerning metabolomics, the metabolite propionylcarnitine (C-3 carnitine) and glycine levels increased upon exposure to AuNPs, which indicates apoptosis. This study further reported the accumulation of GSH in both 5 and 30 nm AuNP-treated cells, which indicates that an anti-oxidative mechanism occurred as a self-defense system against oxidative stress. These results were confirmed using fluorescence microscopy analysis, where the over-expression of Annexin-V and nuclear fragmentation induced by AuNPs were evident, emphasizing that apoptosis occurred.

Omics technology together with complementary methods not only offer a promising tool in nanotoxicology to understand the molecular mechanisms of NP toxicity, but they also enhance the development and design of nano-drugs. For instance, Ali *et al.*^176^ combined MS-based metabolomics and proteomics results through network analysis to better understand the molecular mechanism of AuNR photo-thermal therapy in the human oral squamous cell carcinoma (HSC-3) cell line. The results showed an upregulation in phenylalanine, which is considered an outcome of apoptosis pathways, indicating the good photo-thermal therapy efficiency of the AuNRs. Table 2 summarizes the studies that used the metabolomics technique to assess the effect of AuNPs *in vitro* on different cell lines.

**Table 2:** Summary of AuNP-induced perturbation of metabolic pathways and their biological impact on different cell lines

NP	Size [nm]	Coating	Cell	Dose/exposure time	Analytical platform	Perturbed metabolic pathway	Biological effect	Ref.
AuNRs	15 × 58	CTAB	A549	50 μM	^1^H NMR	Amino acid ↑ in 16HBE nucleosides and nucleotides ↓ in A549	Oxidative stress and cell death in A549	169
			16HBE	12, 24, 48 h				
AuNRs	11 × 42	N/A	GC-2	10 nM	GC-TOF-MS	Glycine ↓ in TM-4	Cell and mitochondrial membrane disruption blood–testis barrier (BTB)	171
			TM-4	24 h		Amino acid		
						Metabolic disruption ↑		
AuNRs	13.2 × 55.7	PSS	A549	50 μM	^1^H NMR	Glucose ↑	Oxidative stress and cell death in A549	177
		PDDAC	16HBE	12, 24, 48 h	GC-FID/MS	Pyruvate ↑		
		PEI				Lactate ↑		
AuNRs	14–16 × 61–78	Phospholipid	MCF-7	0.05, 0.1 nM	LC-MS	Purine	Dysfunction in TCA cycle	178
		PEG		4 h		Pyrimidine	Reduction in glycolytic activity	
						GSH	Imbalance of the redox state	
						Amino acid ↓		
Au	18	Citrate	HepG2	PSS, PVP – 0.25 nM	LC/GC-MS (endo)	Uniform decrease in intracellular metabolite levels	Loss of cell membrane integrity	173
		PSS		Cit – 0.5 nM	^1^H NMR (exo)			
		PVP		3 h				
Au	5	N/A	Caco-2	59 μg mL^−1^	LC-HRMS/MS	5, 30 – amino acid ↑	5 – small molecule biochemistry, cellular assembly and organization, and cellular growth and proliferation	175
	30			72 h		5 – TCA pathway ↓	30 – cellular degeneration and cell morphology	
						30 – glycolysis ↓		
Au	5.67	CeO_2_	HeLa	20 μg mL^−1^	^1^H NMR	CeO_2_ – pyruvate ↑, lactate ↑	CeO_2_ – anaerobic respiration	179
	5.90	Chitosan		24, 48, 72 h		Chitosan – lactate ↓	Chitosan – aerobic respiration	
Au	5.90	Chitosan	HeLa	20 μg mL^−1^	ALSOFAST – ^1^H, ^13^C-HSQC of ^13^C-labeled metabolites	Chitosan – GSH ↑, UDP-NAG ↓, and alteration of glucose metabolism	Chitosan – antioxidant effect	180
	5.65	CeO_2_		48 h		CeO_2_ – no detected metabolites	CeO_2_ – antioxidant effect of this particle is lower and less related with the labelled glucose metabolism	
Au	5.90	Chitosan	RBCs	20 μg mL^−1^	^1^H NMR	Chitosan – reduced GSH ↑	Chitosan – antioxidant effect	181
	5.65	CeO_2_	PMNs	24 h		CeO_2_ – amino acid ↑	CeO_2_ – antioxidant effect of this particle is lower	
			PBMCs				Lower AuChi toxicity compared with AuCeO_2_	
							PMN has higher pronounced antioxidant impact than PBMC	
Au	5	2-Mercapto-1-methylimidazole	SH-SY5Y	100 ng mL^−1^	^1^H NMR	Glutamine, glutamate, leucine, tyrosine, PC/GPC and alanine	Oxidative stress	182
				1, 2, 4, 6 h			Immune response	
							Antioxidant mechanism (restore the initial state)	
Au	20	Citrate	HDFs	200 μM	LC/MS	GSH ↑	Anti-oxidative stress mechanism	172
				4, 8, 24 h				

### 5.2 AgNPs

Silver nanoparticles (AgNPs) have various interesting biological properties and are known for their well-reported antibacterial activity.^183^ They have a wide range of applications including cosmetics, textiles, and biomedical products. Also, their therapeutic application as antiviral and anticancer drugs is expected to be further expanded.^184,185^ Regarding the use of AgNPs as potential drug carriers for cancer therapy from proteogenomic and metabolomic perspectives, the reader is directed to the review by Raja *et al.*^186^ AgNPs have been shown to influence different cells causing apoptosis, lipid peroxidation, and DNA damage.^187–190^

One of the advantages of metabolomics is that it is capable of detecting early biochemical events and metabolic changes even during the absence of a significant cytotoxic response by conventional assays. Carrola *et al.*^191^ studied the effect of citrate-stabilized 30 nm AgNPs on the human epidermis keratinocyte (HaCaT) cell line after 48 h of exposure at two concentrations, *i.e.*, 40 μg mL^−1^ (close to IC_50_ = 38.7 ± 2.5 μg mL^−1^) and 10 μg mL^−1^ (no significant cell viability loss). Using NMR-based metabolomics, the authors observed that most metabolic changes happened at the lower concentration, which allowed the detection of early biochemical events, including upregulated GSH-based antioxidant protection, downregulated tricarboxylic acid (TCA) cycle activity, energy depletion, and cell membrane modification. In a similar study,^192^ NMR metabolomics was used to assess the metabolic effects of two types of coated AgNPs towards the human hepatoma (HepG2) cell line and significant metabolome changes were observed at a subtoxic concentration of AgNPs. These changes include energy production, antioxidant defence system, protein degradation, and lipid metabolism pathways, suggesting that the cells have metabolism-mediated protective mechanisms against AgNPs. In the third study by this group,^193^ they investigated the effect of size and surface chemistry of AgNPs on the metabolic change caused in the HaCaT cell line. The authors used citrate-coated AgNPs with a diameter of 10, 30, and 60 nm, and 30 nm AgNPs coated with citrate, polyethylene glycol (PEG), or bovine serum albumin (BSA). It was found that the largest NPs and the PEG-coated NPs had the least impact on cell metabolism and viability, which is the expected tendency, as mentioned before in Section 3. Furthermore, Carrola *et al.*^194^ used NMR metabolomics to characterize the responses of RAW 264.7 macrophages to subtoxic concentrations of AgNPs (30 nm) and ionic silver (Ag^+^). They observed that the exposure to AgNPs caused a downregulation in intracellular glucose utilization, possibly due to the reprogramming of the TCA cycle towards anaplerotic fuelling and production of anti-inflammatory metabolites. Also, an upregulation in the synthesis of GSH was observed, enabling the cells to control the ROS levels. In contrast, macrophages exposed to Ag^+^ at equivalent subtoxic concentrations showed reduced metabolic activity, lower ability to counterbalance ROS generation, and alterations in membrane lipids. This indicates that the ionic form of silver has a greater effect on the cells and is one of the sources of AgNP cytotoxicity.

**Fig. 12:**
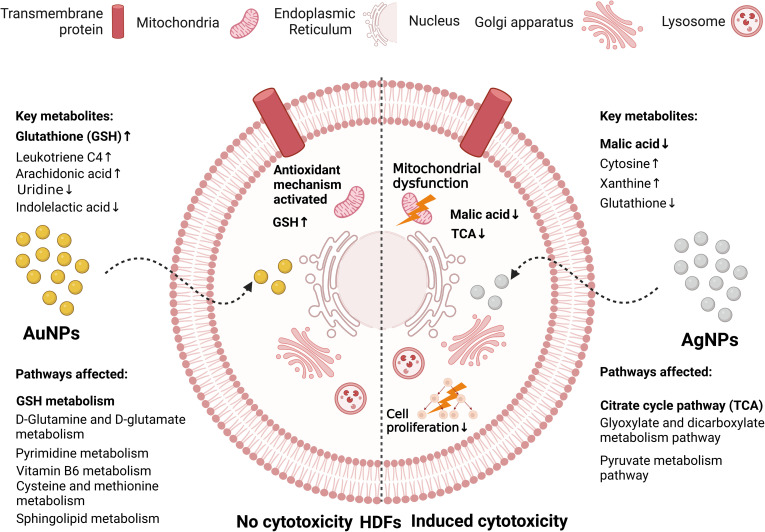
Comparison of the metabolic changes induced due to the interactions between AuNPs or AgNPs with HDFs cells. While AgNPs (Right) induced cytotoxicity in the HDF cells, the effect of AuNPs (Left) was suppressed by an antioxidant mechanism.^172^

Huang *et al.*^172^ compared the effect of AgNPs and AuNPs, and showed that while AuNPs had no cytotoxicity, AgNPs induced grade 1 cytotoxicity after HDF cells were exposed to them for 72 h. Using metabolomics, the citrate cycle pathway was determined to be the key metabolic pathways in the AgNP-treated cells with malic acid as the key metabolite. Thus, the mechanism of AgNP cytotoxicity is by the upregulation of citric acid content, which indicated the inhibition of malic acid synthesis, influencing the production of ATP (mitochondrial dysfunction) and inhibiting cell proliferation, leading to cytotoxicity (see Fig. 12). Conversely, AuNPs were not cytotoxic due to the triggering of the antioxidant defence system by the upregulation of GSH. Kim *et al.*^195^ used high-resolution magic angle spinning (HR-MAS) NMR-based metabolomics to study the cytotoxicity of AgNPs against human Chang liver cells. The authors observed the depletion of GSH, lactate, taurine, and glycine levels, while most amino acids, choline analogues, and pyruvate were upregulated by the AgNPs. It is probable that the downregulation of GSH induced the conversion of lactate and taurine to pyruvate.

The effect of AgNPs was also studied on non-mammalian cells such as yeast and unicellular alga. Babele *et al.*^196^ studied the effect of 1.0 mg L^−1^ of 50–100 nm-sized AgNPs, prepared using aqueous gooseberry extract, on yeast *Saccharomyces cerevisiae* cells. Untargeted ^1^H NMR-based metabolomics revealed a several-fold increase or decrease in the levels of 55 different metabolites, including the ones involved in amino acid metabolism, glycolysis, and tricarboxylic acid (TCA) cycle, organic acids, nucleotide metabolism, urea cycle, and lipids metabolism. The authors noticed a reduced level of GSH, which indicates that oxidative stress occurred, leading to the strong cytotoxicity of AgNPs to the yeast cells. Qu *et al.*^197^ investigated the effect of AgNPs on the performance of *Chlorella vulgaris* F1068 unicellular green alga in phosphorus assimilation (phosphorus removal by algae-based biotechnology). Using MS-based metabolomics, the authors observed the inhibition of algal assimilation. AgNPs disturbed the metabolic responses related to the phosphorus assimilation by reducing the levels of guanine, glutamine, alanine, and aspartic acid and increasing the levels of succinic acid. The NPs also inhibited phospholipid metabolism by the downregulation of glycerol-3-phosphate and myo-inositol and upregulation of serine. Furthermore, GSH metabolism was affected by the NPs, which induced oxidative stress in the alga cells (upregulation of glycine). Cao *et al.*^198^ showed that the effect of AgNPs on *Chlorella pyrenoidosa* can be altered by the number of repeated exposures. In this study, NP single exposure had a greater impact on the *C. pyrenoidosa* metabolome than repeated exposure. Table 3 summarizes the studies that used the metabolomics technique to assess the effect of AgNPs *in vitro* on different cell lines.

**Table 3:** Summary of AgNP-induced perturbation of metabolic pathways and their biological impact on different cell lines

NP	Size [nm]	Coating	Cell	Dose/exposure time	Analytical platform	Perturbed metabolic pathway	Biological effect	Ref.
Ag	20	N/A	HDFs	200 μM	LC/MS	Citric acid ↑	Oxidative stress and cell death	172
				4, 8, 24 h				
Ag	30	Citrate	HaCaT	10, 40 μg mL^−1^	^1^H NMR	GSH ↑	Antioxidant protection	191
				48 h		TCA ↓	Cell membrane modification	
							Energy depletion	
Ag	Cit – 29	Citrate	HepG2	Cit – 6.4, 11.0 μg mL^−1^	^1^H NMR	TCA ↑	Metabolism-mediated protective mechanisms	192
	GS – 33	Biogenic (GS)		GS – 5.4, 14.0 μg mL^−1^		Pyruvate use ↑	Protein degradation	
				24 h		Anaplerotic amino acids ↓		
Ag	10	Citrate	HaCaT	40 mg mL^−1^	^1^H NMR	Glycolysis ↓	Oxidative stress	193
	30	PEG		48 h		Energy production ↓	Largest NP and PEG-NPs have the lowest impact of cell metabolism	
	60	BSA						
Ag	30	Citrate	RAW 264.7	23.2, 35.3 μg mL^−1^	^1^H NMR	Intracellular glucose ↓	ROS/RNS levels control	194
				24 h		TCA		
						GSH ↑		
						Anti-inflammatory metabolites ↑		
Ag	15	N/A	A549	38.6 μg mL^−1^	DIMS	Amino acid ↑	Oxidative stress	199
				1, 6, 24 h		Glycolysis ↓	Apoptosis	
						GSH ↓		
Ag	5–10	N/A	Human Chang liver cell	N/A	HRMAS-^1^H NMR	GSH, lactate, taurine, and glycine ↓	Mitochondria-involved apoptosis	195
						Amino acid, choline analogues, pyruvate ↑	DNA breaks	
							Lipid membrane peroxidation	
							Protein carbonylation	
Ag	69.8	N/A	HT29	25 μg mL^−1^	UPLC Q-TOF MS	Nicotinic acid↑	Mitochondrial dysfunction	200
				12 h		ATP↓	Membrane damage	
							Inhibit cancer proliferation	
Ag	50–100	N/A	Yeast *S. cerevisiae*	1.0 μg mL^−1^	^1^H NMR	Reduced GSH ↑	Oxidative stress	196
				3 h		TCA ↓		
						Glycolysis ↓		
						Amino acid ↓		
						Urea cycle ↓		
Ag	15	N/A	Alga *C. vulgaris* F1068	0.09, 0.2 μg mL^−1^	GC-TOF-MS	Glycerol-3-phosphate ↓	Oxidative stress	197
				148 h		Myo-inositol ↓	Membrane damage	
						Serine ↑	Inhibition of the algal assimilation (66.2% reduction)	
Ag	20	Citrate	Alga *S. obliquus*	1, 10, 100 μg L^−1^	GC-QTOF-MS	Carbohydrates; d-galactose, sucrose, and d-fructose ↑	Growth inhibition	201
				148 h		Amino acids as glycine	Cell wall damage	
						GSH ↑	Oxidative stress	
						TCA interruption		
Ag	20	Citrate	Alga *P. malhamensis*	40.7, 1000 μg L^−1^	LC-MS	Amino acid	Photosynthesis and photorespiration disruption	202
				2, 24 h		TCA	Oxidative stress	
						Nucleotides		
						Fatty acids		
Ag	23.4	PVP	Cyanobacteria *M. aeruginosa*	0.075, 0.15 mg L^−1^	LC-MS	Amino acids; arginine and proline ↑	Cellular stress	203
				96 h		Indole alkaloid biosynthesis ↑	ROS generation	
						Phospholipid metabolism ↑	Damage to photosynthesis and cellular membranes	
Ag	—	N/A	*Chlorella pyrenoidosa*	0.5, 5, and 10 mg L^−1^	LC-MS	Amino acid	Oxidative stress	198
				0–72 h		Carbohydrate	Membrane damage	
				1 or 3 repeated exposure				

### 5.3 TiO_2_ NPs

Micro-titania (titanium oxide, TiO_2_) particles are known as biologically inert in humans, enabling their use in many products such as cosmetics and pharmaceuticals.^204,205^ Nano-titania (TiO_2_ NPs) are also used as additives in many products such as sunscreen products, paints, printing ink, rubber, paper, sugar, cement, toothpaste, film, biomedical ceramics, implanted biomaterials, antimicrobial plastic packaging, and self-cleaning sanitary ceramicss.^206^ However, TiO_2_ NPs can enter the body *via* inhalation, ingestion, and dermal contact and they have been shown to exert significant toxic effects, such as cell metabolic change,^206^ chronic pulmonary inflammation,^207^ and pro-inflammatory effects in cells.^208^ Raja *et al.*^209^ reviewed the microenvironmental influence of TiO_2_ NP-induced mechanical stimuli on tumor cells and showed using the omics analysis that the exposure of cancer cells to TiO_2_ NPs caused gene mutations, protein alterations, and metabolite changes.

Chen *et al.*^210^ observed mitochondrial dysfunction caused by TiO_2_ NPs in a macrophage (RAW) cell line and primary mouse bone marrow-derived macrophages (BMDM) using a combination of metabolomics, lipidomics, and proteomics. The targeted UPLC-MS-based metabolomic analysis revealed a significant upregulation in the production of COX-2 metabolites including PGD2, PGE2, and 15dPGJ2, indicating an inflammatory response in macrophages. The authors also used GC-MS-based metabolic flux analysis, which is a technique that uses MS to track the fate of stable isotope tracers (*e.g.*, ^13^C-glucose and ^15^N-glutamine), allowing the investigation of the contribution of specific metabolic pathways to the prevailing levels of specific metabolites,^211^ to measure the metabolic flux in the tricarboxylic acid (TCA) cycle using ^13^C-labelled glutamine. They observed a downregulation in TCA cycle metabolism and ATP production caused by significant mitochondrial dysfunction after the exposure of macrophages to TiO_2_ NPs. In a similar study, Tucci *et al.*^206^ studied the response of the human keratinocyte HaCaT cell line after exposure to 10–100 nm TiO_2_ NPs and found that the NPs were only present in the phagosomes of the cells without their internalization in any other cytoplasmic organelle. Specifically, “268” metabolites were detected using GC/LC-MS-based metabolomics, of which 85 metabolites were found to be significantly altered at 100 μg mL^−1^ dose of NPs. As stated in other studies, TiO_2_ NPs have shown significant and rapid effects on mitochondrial function by altering energy metabolism and anabolic pathways. However, they did not affect the cell cycle phase distribution or cell death.

Jin *et al.*^212^ used GC/TOFMS-based metabolomics to study the metabolic changes in L929 cells and their corresponding culture media induced by 5 nm-TiO_2_ NPs. At concentrations higher than 100 μg mL^−1^, the NPs caused a depletion in the cellular carbohydrate metabolism (the major biochemical metabolism pathway) after causing energy metabolism disruption, pentose phosphate pathway inhibition, nicotinamide metabolism block, mitochondria damage, and oxidative stress activation. Bo, Jin, Liu *et al.*^213^ again used GC/TOFMS-based metabolomics to study the change in amino acid levels in L929 cells after they were exposed to TiO_2_ NPs. The study revealed that seven metabolic pathways among the regulated pathways were significantly altered including 12 amino acids, *i.e.*, l-α-alanine, β-alanine, glycine, l-aspartate, l-methionine, l-cysteine, glutamate, l-pyroglutamate, l-asparagine, l-glutamine, *S*-adenosyl methionine, and l-lysine.

In dental science, the use of TiO_2_ NPs as an additive to glass ionomer cements is known to improve their mechanical and antibacterial properties. However, the study by Garcia-Contreras *et al.*^214^ showed that these NPs may induce pro-inflammation in human gingival fibroblast (HGF) cells. Nevertheless, the molecular mechanism of the pro-inflammatory action of TiO_2_ NPs on these cells was still unclear. MS metabolomics was used to reveal the mechanism of this pro-inflammatory action by the treatment of HGF cells with IL-1b alone or in combination with TiO_2_ NPs.^215^ A total of 109 metabolites was successfully identified and quantified by CE/TOFMS. Most amino acids levels were downregulated at high concentrations of TiO_2_ NPs, while ophthalmate, α-aminoadipate, kynurenine, and β-alanine were upregulated. The activation of the urea cycle, polyamine, *S*-adenosylmethionine, and GSH synthetic pathways was stronger than that of the other pathways. The intracellular levels of urea cycle metabolites were downregulated significantly in the presence of both IL-1b/TiO_2_ NPs. In conclusion, ornithine was downregulated, which led to an immediate decline in putrescine. That latter is used to synthesize spermidine, which has anti-inflammatory properties. Thus, the reduction of this polyamine level accelerated the inflammation in HGF cells upon exposure to a combination of IL-1b/TiO_2_ NPs.

Kitchin *et al.*^216^ studied the effect of four different TiO_2_ NPs (in addition to two CeO_2_ NPs) on human liver HepG2 cells. Using LC/GC MS-based metabolomics, five out of the six NPs were found to cause a significant downregulation in GSH concentration. The authors observed a decrease in the GSH system in GSH precursors (glutamate and cysteine), GSH itself, and GSH metabolites (the gamma-glutamyl condensation products, glutamine, alanine, valine, 5-oxoproline, and cysteine–GSH). Among the 265 metabolites detected, the reduction in GSH was the largest deregulation. This indicates that the NPs are acting *via* an oxidative stress mode, which is a consistent biochemical effect of NPs.

**Fig. 13:**
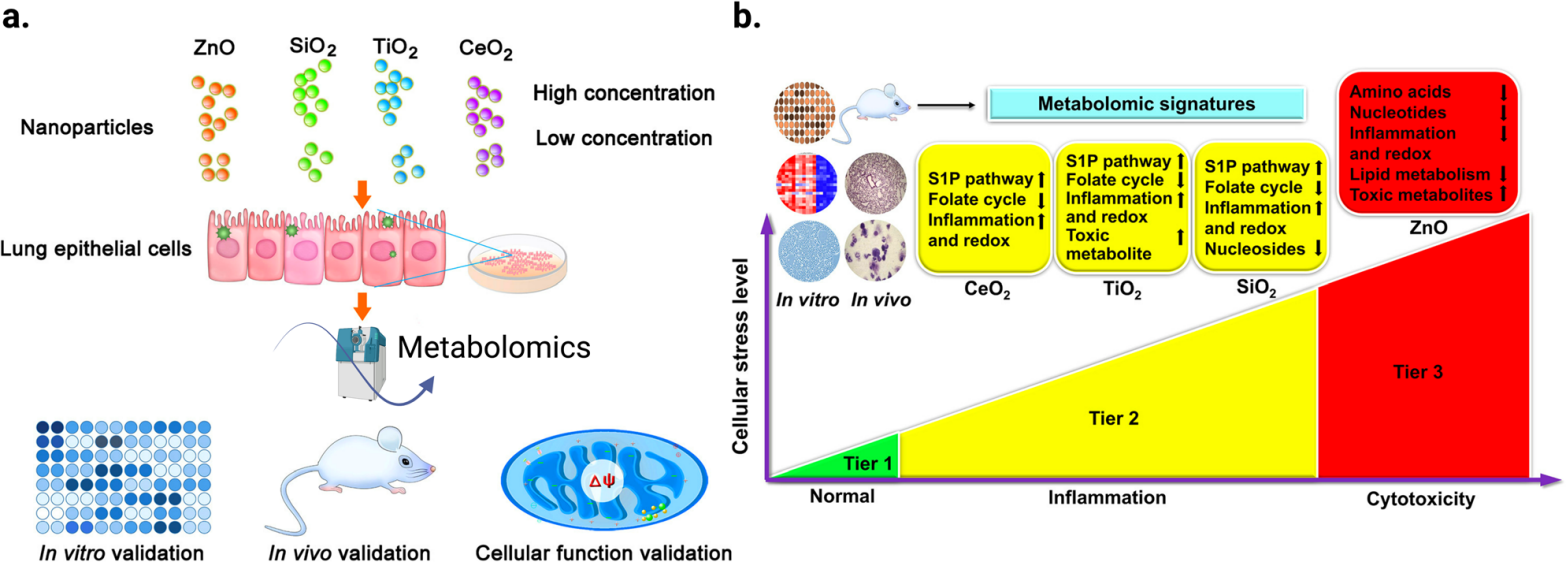
Untargeted metabolomic analysis was used to reveal the effect of exposure of two different doses (12.5 and 25 μg mL^−1^) of ZnO, SiO_2_, TiO_2_, and CeO_2_ NPs on the metabolism of human bronchial epithelial cells (BEAS-2B). (a) Schematic diagram of the study workflow. (b) Hierarchical cellular stress responses based on metabolomics and functional assays. The cells were maintained in the healthy state at the tier 1 stage. At an intermediate level of cellular stress (tier 2), the exposure to SiO_2_, TiO_2_, and CeO_2_ NPs altered several metabolic pathways and induced inflammation. At a high level of cellular stress (tier 3), ZnO NPs significantly affected toxicity and DNA damage related metabolic pathways. Only a short list of significantly altered pathways is presented due to the limited space. Adapted with permission from ref. 217 Copyright 2019, the American Chemical Society.

Metabolomics can help to better understand the transition from *in vitro* to *in vivo* systems of NPs toxicity given that it can be applied in both types of experiment. For example, Cui *et al.*^217^ employed LC-MS-based metabolomics to investigate the effect of four metal oxide NPs, including TiO_2_ NPs, *in vitro* on human bronchial epithelial (BEAS-2B) cell line, and *in vivo* on mouse model after lung exposure. Their study showed that *in vitro* metabolomic findings can effectively reveal the biochemical effects *in vivo*, and that LC-MS-based metabolomics is sensitive enough to detect the tiny metabolomic changes when conventional cytotoxicity assays cannot detect any significant effect. Fig. 13a shows the workflow of this study. BEAS-2B cells were exposed to the four studied NPs, and then the metabolomics experiment was performed *in vitro*. This was followed by validation *in vitro* by enzymatic assays, *in vivo* using a mouse model after lung exposure to respective NPs, and finally by cellular function assays. The TiO_2_ NPs significantly altered the metabolic pathways of sphingosine-1-phosphate, fatty acid oxidation, folate cycle, inflammation/redox, and lipid metabolism, inducing inflammation. In addition, this effect was dose-dependent for some metabolites. Fig. 13b shows the altered metabolites and effect of the four studied metal oxide (MO_*x*_) NPs and their numbers, respectively.

Metabolomics is also applied in many *in vivo* nanotoxicity studies.^218,219^ For instance, Chen *et al.* performed three recent studies of TiO_2_ NP toxicity *in vivo* using MS-based metabolomics, once in rats by feces metabolite analysis,^220^ and then screened for urine^221^ and serum^222^ biomarkers in human workers exposed to these NPs in factories. This group also performed another metabolomics study using rat serum after subchronic oral exposure of TiO_2_ NPs.^223^ Han *et al.*^224^ used MS-based metabolomics to study the influence of TiO_2_ NPs on the fecal metabolome in rats after oral administration for 90 days. Åslund *et al.*^225^ used NMR-based metabolomics to assess the effects of 5 nm-TiO_2_ NPs on *Eisenia fetida* earthworms and observed metabolic changes related to oxidative stress. Eight years later, Zhu *et al.*^226^ used transcriptomics besides metabolomics to investigate the same effect of TiO_2_ NPs on the same earthworm and noticed that the antioxidant system and metabolic profiles of the earthworms were significantly affected. Ratnasekhar *et al.*^227^ used MS-based metabolomics to investigate the effects of TiO_2_ NPs on the soil nematode *Caenorhabditis elegans*. The results indicated the disruption of the tricarboxylic acid (TCA) cycle, arachidonic acid metabolism, and glyoxylate dicarboxylate metabolism pathways. For more about the *in vivo* metabolic effects of NPs including Ag, TiO_2_, and carbon-based NPs on organisms (plants, aquatic, and terrestrial invertebrates), the reader is kindly referred to the chapter by Farré and Jha.^165^

Metabolomics reveals the global responses that cannot be observed by conventional toxicity endpoints, leading to an effective assessment of the effects of NPs in the environment, *in vivo*, and *in vitro*. Metabolomics has also been used to reveal the metabolite corona that is surrounding TiO_2_ NPs.^228,229^Table 4 summarizes the studies that used the metabolomics technique to assess the effect of TiO_2_ NPs *in vitro* on different cells.

**Table 4:** Summary of TiO_2_ NP-induced perturbation of metabolic pathways and their biological impact on different cells

NP	Size [nm]	Coating	Cell	Dose/exposure time	Analytical platform	Perturbed metabolic pathway	Biological effect	Ref.
TiO_2_	10	N/A	RAW264.7	10, 100 μg mL^−1^	UPLC-MS	COX-2; PGD_2_, PGE_2_, 15 d-PGJ_2_ ↑	Mitochondrial dysfunction	210
			BMDM	24 h	GC-MS for ^13^C-labelled glutamine (flux analysis)	TCA ↓	Inflammatory response	
						ATP ↓		
TiO_2_	10–100	N/A	HaCaT	5, 50, 100 μg mL^−1^	GC-MS	Acetyl-CoA	Oxidative stress	206
				24 h	LC/MS/MS	GSH ↓	Mitochondrial function disruption	
						Acetyl-carnitine		
						Glycolysis ↓		
						Pentose phosphate pathway ↓		
						Nucleotide ↓		
TiO_2_	N/A	N/A	L929	30 μg mL^−1^	GC-TOF-MS	Carbohydrate metabolism and TCA ↓	Metabolism changes	150
				48 h		Glycolysis ↓		
						Fatty acid ↓		
						Purine metabolism ↓		
TiO_2_	5	N/A	L929	100, 200 μg mL^−1^	GC-TOF-MS	Carbohydrate metabolism and TCA ↓	Mitochondria damage	212
				48 h		Pentose phosphate pathway↓	Oxidative stress	
						Nicotinamide metabolism block		
TiO_2_	5	N/A	L929	100 μg mL^−1^	GC-TOF-MS	Amino acid ↑	Oxidative stress	213
				48 h		Carbohydrate ↑	Energy damage	
						Nucleotide ↑	Inhibition of DNA and RNA synthesis	
TiO_2_	18	N/A	HGF	0.2, 0.8, 3.2 mM + IL-β 3 ng mL^−1^	CE-TOF-MS	Amino acid ↓	Inflammation	215
				24 h		Urea cycle ↓		
						GSH synthesis ↓		
						Polyamine ↓		
TiO_2_	8–142	N/A	HepG2	3, 30 μg mL^−1^	GC-MS	GSH ↓	Oxidative stress	216
				72 h	UPLC/MS/MS	Fatty acid		
TiO_2_	21	N/A	BEAS-2B	12.5, 25 μg mL^−1^	LC-MS	S1P pathway ↑	Inflammation	217
				6 h		Folate cycle ↑	Oxidative stress	
						Fatty acid oxidation	DNA damage	
						GSH ↓		
						Lipid ↑		
TiO_2_	21	N/A	RLE-6TN	0.1, 1, 10 μg cm^−2^	HPLC-MS/MS	Lipids ↑	Oxidative stress	231
				24 h	FIA-MS/MS	Amino acid		
						Biogenic amines		
TiO_2_	21	N/A	RLE-6TN	1, 2.5, 5, 10, μg cm^−2^	HPLC-MS	Amino acid	Oxidative stress	230
			NR8383	24, 48 h		Biogenic amine		
						Lipid		
TiO_2_	21	N/A	RLE-6TN	0.1–50 μg cm^−2^	HPLC-MS	GSH	Oxidative stress	232
			NR8383	24, 48 h		Amino acid		
						Biogenic amine		
						Lipid		
TiO_2_	42	N/A	*E. coli*	2.5–10 μg mL^−1^	GC-TOF-MS	Polyamine; putrescine ↓	Oxidative stress	233
				3 h		Amino acid; glycine ↑		
TiO_2_	8–37	N/A	*E. coli*	10, 100 ppm	^1^H NMR	TCA ↑	Cell membrane damage	234
				3 h		Amino acid ↑	Oxidative stress	
						ATP ↑		
						Fatty acid ↑		
						Polyamine; putrescine ↑		
TiO_2_	30	N/A	*P. polycephalum* macroplasmodium	9, 15 18 mg mL^−1^	GC-MS	Amino acid	Oxidative stress	235
				72 h		GSH↑	ROS imbalance	
						Nucleotide		
						Polyamine		
						Carbohydrate		
TiO_2_/CdS/ZnS	10/30/40	N/A	*Bacillus subtilis*	0.1953, 3.125 mg mL^−1^	LC/MS	Lipid ↓	Membrane damage	236
						Biomolecules synthesis ↓	ROS generation	
						ATP ↓		

### 5.4 SiO_2_ NPs

The annual global production of SiO_2_ NPs is reported to exceed 1.5 million tons, making SiO_2_ NPs one of the most widely used NPs in the industrial manufacturing, drug delivery, cancer therapy, and biotechnological fields.^39^ This widespread is due to their biocompatibility, stability, and other unique properties compared with their bulk.^237^

Although SiO_2_ NPs have been shown to have different cytotoxic effects on cells, the molecular mechanism of this cytotoxicity still needs to be explored using novel analytical techniques, such as metabolomics. Huang *et al.*^238^ used MS-based metabolomics to reveal the molecular information of the effect of SiO_2_ NPs on the human fetal lung fibroblast MRC-5 cell line. The authors observed NP dose-dependent changes in the metabolic profiles of the cells. As the dose increased, there was a downregulation in the amino acid and GSH levels together with an upregulation in urea and phospholipid concentrations, causing oxidative stress and energy metabolism disturbance. Feng *et al.*^237^ used NMR-based metabolomics to study the effects of 0.01 or 1.0 mg mL^−1^ of hydrophilic SiO_2_ NPs on the human cervical adenocarcinoma (HeLa) cell line. They studied both the intracellular and extracellular metabolome changes. In the early stage of NP exposure, no clear dose-effect of the HeLa cell metabolome was observed, which implied that the cellular stress-response regulated the metabolic variations in the HeLa cells. Afterwards, the NPs induced cell membrane modification, catabolism of carbohydrate and protein, and a stress response. The toxicological effects induced by high-dosage SiO_2_ NPs could be derived from the elevated levels of ATP and ADP, the utilization of glucose and amino acids and the production of metabolic end-products such as glutamate, glycine, lysine, methionine, phenylalanine, and valine. Irfan *et al.*^239^ used conventional assays and NMR-based extracellular metabolomics to study the effect of fumed SiO_2_ NPs on human lung A549 cells. The authors observed an upregulation in the extracellular glucose, lactate, phenylalanine, histidine, and tyrosine levels in a time- and NP dose-dependent manner. There was also an increase in intracellular ROS and cell membrane damage at 4 h and a loss of cell viability after 48 h observed by conventional assays.

**Fig. 14:**
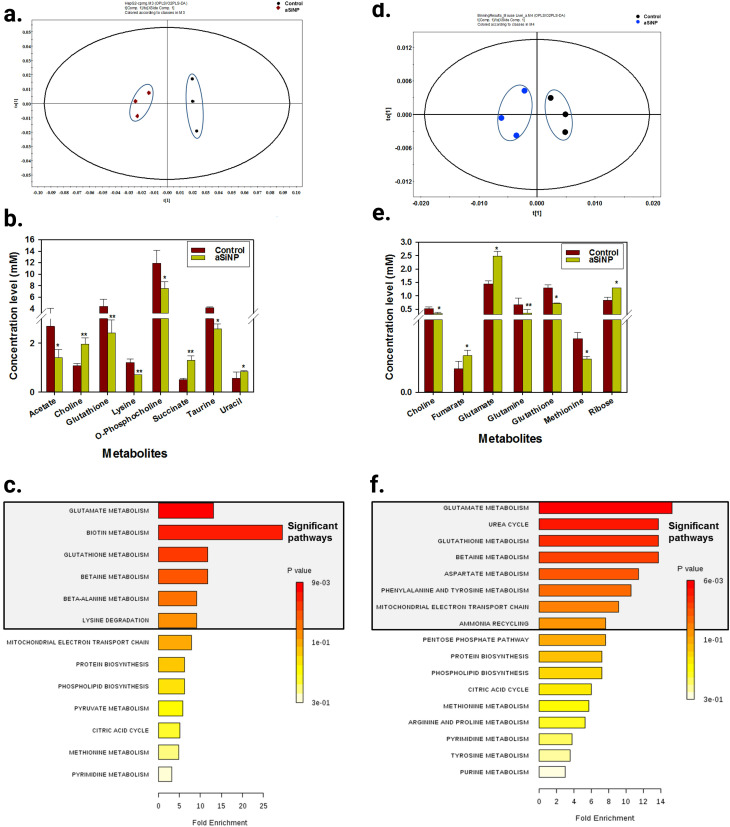
Global metabolomics and pathway analysis in aSiO_2_ NP-exposed HepG2 cells (a–c) and Institute for Cancer Research (ICR) mice (d–f). OPLS-DA score plot from the NMR spectra of metabolomes from HepG2 cells treated with 100 mg L^−1^ aSiO_2_ NPs for 24 h (a) and from ICR mice liver treated with 50 mg kg^−1^ aSiO_2_ NPs for 24 h. Selected significantly altered global metabolites level in HepG2 cells (b) and in ICR mice liver (e) after treatment with the NPs. Pathway-based enrichment analysis performed by MetaboAnalyst 3.0 with significant altered metabolites (>1.5 fold) in HepG2 cells (c) and ICR mice liver (f) after treatment with the NPs. This figure has been reproduced from ref. 240 with permission from Elsevier B.V., Copyright 2018.

A few metabolomics studies compared the *in vitro* and *in vivo* outcomes of SiO_2_ NP treatments. For instance, Chatterjee *et al.*^240^ used NMR-based untargeted-metabolomics to study the effect of amorphous SiO_2_ NPs on the human hepatoma HepG2 cell line and mice liver (Fig. 14). Firstly, this study determined the altered metabolites in the cells and mice liver using OPLS-DA analysis (Fig. 14a and d, respectively). Subsequently, the selected significantly altered metabolites were determined (Fig. 14b and e), followed by pathway analysis using the MetaboAnalyst 3.0 software (Fig. 14c and f). In both *in vitro* and *in vivo* systems, the perturbation of GSH metabolism and the depletion of the GSH pool were detected after aSiO_2_ NP treatment. Moreover, the *in vitro* results were further supported by the *in vivo* data, specifically for metabolite profiling and pathway analysis, were there were 8 common altered metabolic pathways in the two systems. This study revealed that the major causes of aSiO_2_ NP-mediated hepatotoxicity were the suppression of GSH metabolism and oxidative stress. In a similar study, Bannuscher *et al.*^230^ studied the responses of rat lung epithelial cells (RLE-6TN) and alveolar macrophages (NR8383) (*in vitro*) to four well-selected SiO_2_ NPs, differing in structure, size, and surface charge, and compared the results to *in vivo* responses in rat lung tissues. The authors observed a cell-specific time- and concentration-dependent changes *in vitro* and identified several biomarker candidates such as Asp, Asn, Ser, Pro, spermidine, putrescine, and LysoPCaC16:1 *in vitro*, and then verified them *in vivo*.

It was proven that SiO_2_ NP exposure inevitably induces damage to the respiratory system, however, knowledge of its mode of action and metabolic interactions with the cells is limited. Zhao *et al.*^241^ performed a study to reveal the molecular information of the metabolic responses of the lung bronchial epithelial BEAS2B cell line after SiO_2_ NP exposure, using MS-based metabolomics. They revealed that even with low cytotoxicity, SiO_2_ NPs still caused global metabolism disruption. Specifically, five metabolic pathways were significantly perturbed; in particular, oxidative stress, as confirmed by GSH depletion, mitochondrial dysfunction-related GSH metabolism, and pantothenate and coenzyme A (CoA) biosynthesis. The identified key metabolites were GSH, glycine, beta-alanine, cysteine, cysteinyl-glycine, and pantothenic acid. Oxidative DNA damage and cell membrane disintegration were detected by observing elevated 8-oxo-2′-deoxyguanosine (8-OdG) and decreased phospholipids levels.

Several studies compared the effect of SiO_2_ NPs on cells to other NPs using metabolomics and other omics techniques. For example, Karkossa *et al.*^231^ used targeted metabolomics and global proteomics to compare the effect of SiO_2_ NPs with different particle sizes, surface charges, and hydrophobicity to the effect of TiO_2_, graphene oxide (GO), phthalocyanine blue, phthalocyanine green, and Mn_2_O_3_ NPs on RLE-6TN alveolar epithelial cells. Alternatively, Cui *et al.*^217^ used MS-based metabolomics to reveal the significantly altered metabolites and metabolic pathways in human bronchial epithelial cells and a mouse model exposed to four different types of metal oxide NPs (SiO_2_, ZnO, TiO_2_, and CeO_2_) at both high (25 μg mL^−1^) and low (12.5 μg mL^−1^) doses (see Fig. 13). Table 5 summarizes the studies that used metabolomics technique to assess the effect of SiO_2_ NPs *in vitro* on different cells.

**Table 5:** Summary of SiO_2_ NP-induced perturbation of metabolic pathways and their biological impact on different cells

NP	Size [nm]	Coating	Cell	Dose/exposure time	Analytical platform	Perturbed metabolic pathway	Biological effect	Ref.
SiO_2_	45	FBS	MRC-5	2.5, 10, 40, 80 μg mL^−1^	GC-MS	Amino acid ↓	Oxidative stress	238
		Tris(2,2′-bipyridyl)-dichlororuthenium(ii) hexahydrate		24 h	LC-MS	GSH↓	Energy metabolism disturbance	
						Urea cycle ↑	Membrane damage	
						Phospholipid ↑	Cell death	
SiO_2_	20	N/A	HeLa	0.01, 1 mg mL^−1^	HRMAS ^1^H NMR	Lipid ↑	Catabolism of carbohydrate and protein	237
				6, 48 h		Carbohydrate and protein metabolism	Cell membrane modification	
						Amino acid	Oxidative stress	
SiO_2_	7–14	N/A	A549	10, 25, 50, 100 μg mL^−1^	^1^H NMR	Glucose ↑	Oxidative stress	239
				4, 12, 24, 48 h		Lactate ↑		
						Phenylalanine ↑		
SiO_2_	57.7	N/A	BEAS-2B	2, 10, 50 μg mL^−1^	UPLC-MS	GSH ↓	Oxidative stress	241
				24 h		8-OdG ↑	DNA damage	
						Phospholipid ↓	Mitochondrial dysfunction	
						ATP ↓	Cell membrane dis-integrity	
						NRF2 ↓		
						Pantothenate and CoA biosynthesis		
SiO_2_	16	N/A	BEAS-2B	12.5, 25 μg mL^−1^	LC-MS	S1P pathway ↑	Inflammation	217
				6 h		Folate cycle ↑	Oxidative stress	
						Fatty acid oxidation	DNA damage	
						Lipid		
SiO_2_	10–30	N/A	HepG2	30 μg mL^−1^	GC-MS	Lipid ↑	Oxidative stress	242
				72 h	LC-MS	GSH ↓		
SiO_2_	20–50	N/A	HepG2	100 μg mL^−1^	^1^H NMR	GSH ↓	Oxidative stress	240
				24 h		Acetate ↓		
						Choline ↑		
						Biotin metabolism		
SiO_2_	15, 15, 8, 40	None, amino, none, none	RLE-6TN	1, 2.5, 5, 10, 50 μg cm^−2^	HPLC-MS	Amino acid	Oxidative stress	230
			NR8383	24, 48 h		Biogenic amine		
						Lipid		
SiO_2_	15, 15, 15, 40, 8, 8, 8	None, phosphate, amino, none, none, 2% TMS, 3% TMS	RLE-6TN	10 μg cm^−2^	HPLC-MS/MS	Amino acid	Oxidative stress	231
				24 h	FIA-MS/MS	Biogenic amine	Apoptosis	
						Lipid		
SiO_2_	8, 15, 40	N/A	NR8383	2.5, 5, 10 μg cm^−2^	HPLC-MS	Amino acid	Oxidative stress	243
				24 h		Biogenic amine	Mitochondrial dysfunction	
						Phosphatidylcholine	DNA damage	
							Cell death	
SiO_2_	8, 15, 15, 40	None, amino, none, none	RLE-6TN	1–50 μg cm^−2^	HPLC-MS	GSH	Oxidative stress	230
			NR8383	24, 48 h		Amino acid		
						Biogenic amine		
						Lipid		
SiO_2_	100–125	N/A	RAW 264.7	10, 500 μg mL^−1^	^1^H NMR	Glycolysis ↑	Inflammation	244
				24, 48, 72 h		Lactate ↑		
						ATP ↓		
						TCA		
SiO_2_	58	N/A	L-02	25, 50 μg mL^−1^	UHPLC-MS	Adenine ↑	Oxidative stress	245
				24 h		d-Glucose ↑	Cell death	
						GSH ↓	Cell viability	
						Adenosine triphosphate ↓		

### 5.5 ZnO NPs

Zinc oxide NPs are gaining increasing attention due to their unique properties, especially their optical and electronic properties. Also, they can be prepared using a variety of methods and in a range of different morphologies.^246^ This makes them the third highest global production volume among metal-containing NMs^247^ and excellent for a broad range of applications, including optoelectronic devices (light-emitting diodes (LEDs), laser diodes, solar cells, and photodetectors), electronic devices (transistors),^246^ and active compounds in sunscreens, drug delivery, biomedical engineering, food additives, and cosmetics.^248^ It was shown that human exposure to these engineered NPs can cause health problems for both consumers and industry workers, making it important to further investigate in their toxicity and improve their safety when they are used and produced.^217,249^

**Fig. 15:**
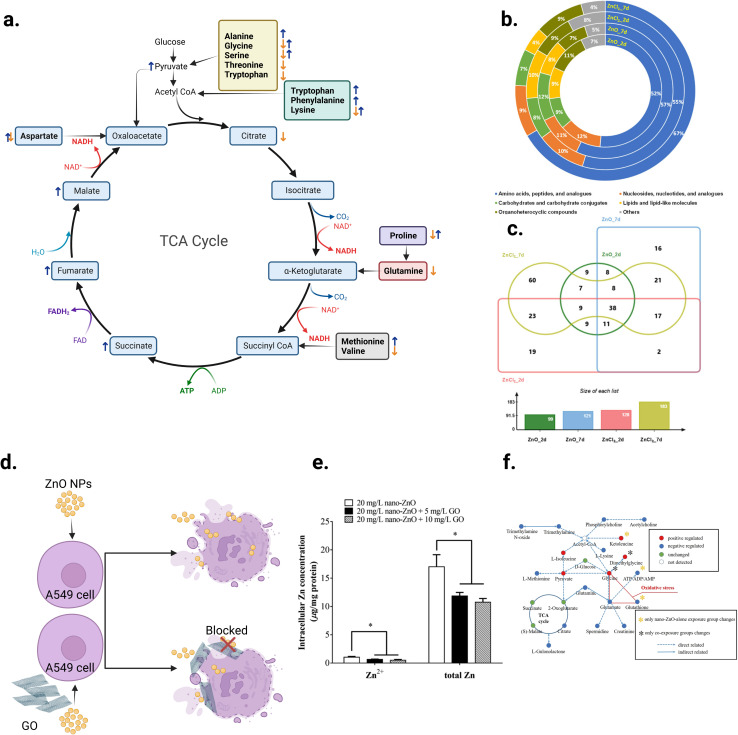
[(a) Comparison of ZnO NPs with PS NPs].^247^ (a) Summary comparing the relevant metabolic responses of BEAS-2B cells to ZnO and PS_LD NP exposure. Arrow in orange or blue represents a significant increase or decrease in the ZnO or PS_LD NP exposure, respectively, compared with the non-treated groups. This figure is adapted with permission from ref. 247 Copyright 2019, Taylor & Francis. [(b and c) Comparison of ZnO NPs with Zn^2+^].^254^ (b) Proportion of significantly changed metabolites in different categories after 2 (2 d) and 7 (7 d) days exposure to ZnO NPs and ZnCl_2_. (c) Edwards–Venn diagram of the total number of significantly changed metabolites. The total numbers of significantly changed metabolites in ZnO_2 d (d = days), ZnO_7 d, ZnCl_2__2 d, and ZnCl_2__7 d groups were 99, 121, 128, and 183, respectively. The altered metabolites were obtained by conducting PLS-DA analyses for each Zn-exposed group *vs.* the matched control group (VIP > 1 and *p* < 0.05). Note: the metabolites identified from the positive ion mode and negative ion mode were merged together. (b and c) Are reprinted with permission from ref. 254 Copyright 2020, the American Chemical Society. [(d–f) Comparison of ZnO NPs solo exposure to their co-exposure with GO].^257^ (d) Schematic illustration of A549 cells ZnO NPs solo exposure *vs.* co-exposure with GO. The GO sheets reduce the cytotoxicity of ZnO NPs by blocking their internalization into the A549 cells. (e) Influence of GO on the bioavailability of ZnO NPs. The Zn concentrations were normalized by the protein concentrations. All data expressed as the mean ± SD. All differences were identified by one-way ANOVA followed by Tukey post hoc test. * Indicates *p*-value. (f) Interaction network of metabolites in ZnO NPs solo and co-exposure groups. (d–f) Are reproduced from ref. 257 with permission from The Royal Society of Chemistry, Copyright 2019.

The respiratory tract is the primary route of exposure to airborne NPs such as ZnO. Thus, it is common to use the bronchial epithelial BEAS-2B cell line as an *in vitro* model to study the toxicity of these NPs. For instance, Lim *et al.*^247^ used this cell line to perform an MS-based metabolomics study to reveal the effect of ZnO NPs on the respiratory system. The authors revealed ROS-mediated cell death associated with mitochondrial dysfunction and interference in regulating energy metabolism. This was concluded after observing a significant decrease in the levels of amino acids (valine, tryptophan, lysine, proline, threonine, glycine, serine, glutamic acid, and aspartic acid) and TCA intermediate metabolites (citrate) (Fig. 15a). These results indicate that ZnO NPs can be seriously harmful to human health if they were inhaled.

Although Zn is a key micronutrient for plants, a high dose of this metal is toxic to plants either in the nano or other forms. Salehi *et al.*^250^ used UHPLC-QTOF metabolomics to study the effect of ZnO NPs and bulk ZnSO_4_ on bean plants (*Phaseolus vulgaris* L). The results indicated the unique NP-related toxic effects of ZnO in beans compared to the ionic forms of Zn. Two similar studies of the effect of ZnO NPs have been done on tomato and cucumber.^251,252^ Wan *et al.*^253^ performed a metabolomics analysis to reveal the effect of ZnO NPs on salt tolerance in the *Sophora alopecuroides* plant. Moreover, He *et al.*^254^ elucidated toxicodynamic differences at the molecular scale between ZnO NPs and ZnCl_2_ in *Enchytraeus crypticus*, a model species in soil ecotoxicology, using non-targeted metabolomics. They found that the number of altered metabolites after Zn^2+^ exposure was larger than the number of altered metabolites after ZnO NP exposure, indicating the higher toxicity of the Zn ionic form (Fig. 15b and c). For more information about nanotechnology in agriculture and the effect of metallic-, metal oxide-, and carbon-based-NPs on plants, the reader is advised to read the review by Paramo *et al.*^255^ and review by Majumdar *et al.*^256^

The toxic effects of a NP may be reduced by applying co-exposure with another NP. For instance, Wu *et al.*^257^ studied the combined effects of graphene oxide (GO) and ZnO NPs on human A549 cells using NMR-based metabolomics. PLS-DA analysis showed that the control and GO-alone exposure groups overlapped, indicating a low effect of 10 mg L^−1^ GO on the metabolome profiles. In contrast, ZnO NP-alone exposure significantly altered the metabolome profiles in A549 cells. A total of 14 altered metabolites was shared in the ZnO NP-alone and the co-exposure with GO groups. However, the levels of fold changes of the 14 shared metabolites were lower in the co-exposure group than that in the ZnO NP-alone group. This tendency indicates that GO alleviated the toxicity induced by ZnO NPs in the cellular metabolism by reducing or blocking their internalization in the cells (Fig. 15d–f). Table 6 summarizes the studies that used metabolomics technique to assess the effect of ZnO NPs *in vitro* on different cells.

**Table 6:** Summary of ZnO NP-induced perturbation of metabolic pathways and their biological impact on different cells

NP	Size [nm]	Coating	Cell	Dose/exposure time	Analytical platform	Perturbed metabolic pathway	Biological effect	Ref.
ZnO	60	N/A	BEAS-2B	10 μg mL^−1^	HPLC-MS/MS	Amino acid ↓	Mitochondrial dysfunction	247
				24 h	GC-MS/MS	TCA ↓	Cell death	
							ROS	
ZnO	22.6	N/A	BEAS-2B	12.5, 25 μg mL^−1^	LC-MS	Amino acid ↓	Inflammation	217
				6 h		Nucleotides ↓	Oxidative stress	
						Lipid ↓	DNA damage	
						Toxic metabolites ↑	High cytotoxicity	
ZnO-GO	ZnO – 50	N/A	A549	ZnO – 20 μg mL^−1^	^1^H NMR	TCA ↓	Membrane damage	257
				GO – 10 μg mL^−1^		GSH ↓	Oxidative stress	
				24 h		Choline	Energy metabolism disruption	
						Amino acid	GO reduced the impact of nano-ZnO	
						Carbohydrate		
ZnO	42	None	A549	10, 15 μg mL^−1^	DIMS	GSH ↓	Oxidative stress	199
	34	Triethoxycaprylsilane		1, 6, 24 h		Amino acid	Apoptosis	
ZnO	71	N/A	*E. coli*	0.025–0.2 μg mL^−1^	GC-TOF-MS	Amino acid; glycine ↑	Oxidative stress	233
				3 h				
ZnO	<70	N/A	Yeast *S. cerevisiae*	10 μg mL^−1^	^1^H NMR	Amino acid	DNA and protein damage	258
			BY4741	3 h		TCA ↓	Oxidative stress	
						GSH ↓	Antioxidation	
						Glycolysis ↓	Energy metabolism disruption	
						Fatty acid ↓		
						Purine and pyrimidine ↓		

### 5.6 Other metal- and metal oxide-NPs

#### 5.6.1 Cobalt ferrite (CoFe_2_O_4_) NPs

Cobalt ferrite (CoFe_2_O_4_) NPs have interesting properties, such as mechanical hardness, excellent chemical stability, high anisotropy, superparamagnetism, and coercivity.^259^ Oliveira *et al.*^260^ studied the cytotoxic effect, cellular uptake, and metabolomic effect of CoFe_2_O_4_ NPs on the HeLa and HaCaT cell lines. This study revealed, using NMR-based metabolomics, that although the uptake of NPs at 2 mg mL^−1^ caused low cytotoxicity, it significantly impacted the cell metabolism. Both cell lines shared stress-related metabolic changes such as upregulation in alanine and creatine. A downregulation in fumarate level was present in HeLa cells treated with the NPs. Given that this metabolite is associated with cell proliferation and tumor growth, it was concluded that CoFe_2_O_4_ NPs can inhibit tumorigenesis.

#### 5.6.2 Copper oxide (CuO) NPs

Copper oxide (CuO) NPs have been used in heat transfer fluids, semiconductors, and intrauterine contraceptive devices.^261^ Human exposure to CuO NPs is rapidly increasing, and thus reliable toxicity test systems are urgently needed. It was shown that CuO NPs are more toxic than their microparticles (MPs). To reveal the mechanism of this toxicity, Murgia *et al.*^262^ used MS-based metabolomics to the study the effect of CuO micro- and nano-particles against human bone marrow mesenchymal stem cells (hBMMSCs). It was found that the MPs increased the levels of serine, glyceric acid, and succinic acid, while glutamine was the only discriminant metabolite for the class of samples treated with NPs. This proves that ROS formation is the active mode of action in NP treatment, providing the first step toward the understanding of the mechanism of toxicity of CuO NP-treated cells. Wang *et al.*^263^ compared the effect of CuO NPs, MPs, and Cu ions on microalga *Chlorella vulgaris* after 5 days exposure using global metabolomics. A total of 75 differentiated metabolites was identified. Most metabolic pathways perturbed after CuO NP exposure were shared by that after CuO MP and Cu ion exposure. Only one difference between metabolic responses to particles and that to ions was observed, which is the accumulation of fatty acid oxidation products, *i.e.*, particles caused higher fold changes at 1 mg L^−1^ and lower fold changes at 10 mg L^−1^ compared with ions. This indicates the significant role of dissolved Cu ions on the toxicity of CuO NPs and MPs. Kruszka *et al.*^264^ compared the effect of Cu and CuO NPs on the secondary metabolism of *Hypericum perforatum* L. cell suspension cultures and found that metal NPs induce higher metabolic changes than their counterpart metal oxide NPs. Table 7 summarizes the studies that used the metabolomics technique to assess the effect of other metal/metal oxide NPs *in vitro* on different cells.

**Table 7:** Summary of metal/oxide NM NP-induced perturbation of metabolic pathways and their biological impact on different cells

NP	Size [nm]	Coating	Cell	Dose/exposure time	Analytical platform	Perturbed metabolic pathway	Biological effect	Ref.
CuO	30	N/A	hBMMSCs	2.51 μg mL^−1^	GC-MS	Serine	ROS formation	262
				48 h		Glyceric acid		
						Succinic acid		
						Glutamine		
CuO	20–80	N/A	HepG2	3 μg mL^−1^	GC-MLC-MS	*S*-Adenosylhomocysteine ↑	Oxidative stress	216
				72 h		Lysolipids ↑		
						Sphingolipids ↑		
						*S*-Adenosylmethionine ↓		
						GSH ↓		
CuO	28	N/A	A549	10 μg mL^−1^	HPLC-MS	MNA ↑	Oxidative stress	265
				0, 1, 3, 6, 12, 24 h		GSH ↓	Hypertonic stress	
						GPC ↑	Apoptosis	
						MTA ↑		
						Amino acid ↑		
CuO	<50	N/A	HCT-116	2.5, 5 μg mL^−1^	LC-QToF-MS	Triacylglycerols ↑	Autophagy	266
				24 h		Phosphatidylcholines ↑	Oxidative stress	
						Ceramides ↑		
						GSSG ↑		
CuO	15–50	N/A	Alga *C. vulgaris*	1, 10 μg mL^−1^	LC-QToF-MS	Fatty acid ↑	Oxidative stress	263
				120 h		GSH ↓	Osmotic stress	
						Phosphatidylcholines ↓	ROS formation	
						Phosphatidylglycerols ↑	Membrane damage	
CeO_2_	5–50	N/A	HepG2	30, 100 μg mL^−1^	GC-MS	*S*-Adenosylhomocysteine ↑	Oxidative stress	242
				72 h	LC-MS	Lipids ↑		
						UDP-glucuronate ↓		
						*S*-Adenosylmethionine ↓		
						GSH ↓		
CeO_2_	8	N/A	HepG2	3, 30 μg mL^−1^	GC-MS	GSH ↓	Oxidative stress	216
	58			72 h	UPLC/MS/MS	iNOS ↓		
						Lipids ↑		
						Fatty acid		
CeO_2_	4.7	None, or Zr doped	A549	128 μg mL^−1^	DIMS	Cysteine ↑	Oxidative stress	207
	28			1, 6, 24 h		γ-Glutamylcysteine ↑	Apoptosis	
CeO_2_	50 × 6.7	N/A	BEAS-2B	12.5, 25 μg mL^−1^	LC-MS	S1P pathway ↑	Inflammation	217
				6 h		Folate cycle ↑	Oxidative stress	
						GSH ↓	DNA damage	
						Lipid ↑		
CeO_2_	210	N/A	HuH-7	100 μg mL^−1^	HPLC-MS	Histamine ↑	Genotoxicity	267
				24 h		Hydroxyproline ↑		
						Proline ↓		
CeO_2_	4–5	PVP	Alga *C. reinhardtii*	0.029, 0.144, 0.7280, 400, 2000, 10 000 μg L^−1^	LTQ-FT-MS	Only at 10 000 μg L^−1^	Photosynthesis decrease	268
				72 h		Pyruvate ↑	Energy metabolism disruption	
						GSH ↑		
						Purine ↑		
						Pyrimidine ↓		
CoFe_2_O_4_	27	N/A	HeLa	2 mg mL^−1^	^1^H NMR	Alanine ↑	Metabolic change	260
			HaCaT	24 h		Creatine ↑	HeLa – inhibition of tumorigenesis	
						GSH ↑		
						HeLa – fumarate, lactate ↓		
CoFe_2_O_4_	Core-9	(RITC)-SiO_2_	HEK293	0.1, 1.0 μg mL^−1^	GC-MS/MS	Glucose ↓	ROS generation	30
	Full-50			12 h		Amino acid	Glucose metabolic dysfunction	
						Fatty acid		
						Polyamine		
						Organic acid		
CoFe_2_O_4_	Core-9	(RITC)-SiO_2_	HEK293	1.0 μg mL^−1^	GC-MS/MS	Glutamic acid ↑	Mitochondrial damage	156
	Full-50					Krebs cycle	ROS generation	
						ATP ↓		
CoFe_2_O_4_	Core-9	(RITC)-SiO_2_	HEK293	1.0 μg mL^−1^	GC-MS/MS	Lipid ↓	Membrane fluidity decreased	269
	Full-50					ATP ↓	Cell movement decreased	
CoFe_2_O_4_	Core-9	(RITC)-SiO_2_	BV2 murine microglial cells	0.1, 1.0 μg mL^−1^	GC-MS/MS	GSH	ROS generation	270
	Full-50			12 h		TCA	Inflammatory response	
Mn_2_O_3_	5	N/A	RLE-6TN	10 μg cm^−2^	HPLC-MS/MS	No significant metabolic changes	Oxidative stress	231
				24 h	FIA-MS/MS		Apoptosis	
							High cytotoxicity	
Mn_2_O_3_	50	N/A	NR8383	2.5, 5, 10 μg cm^−2^	HPLC-MS	Amino acid	Oxidative stress	243
				24 h		Biogenic amine	Mitochondrial dysfunction	
						Phosphatidylcholine	DNA damage & cell death	
Al_2_O_3_	64	N/A	HBE	100, 500 μg mL^−1^	GC/TOF-MS	d-Glutamic acid ↑	Mitochondria-dependent apoptosis	271
				24 h		Succinic acid ↑	Oxidative stress	
						3-Methylhistidine ↓	ROS generation	
						TCA ↓		
γ-Al_2_O_3_	[20–30 × 20–30]	N/A	ASTs	125 μg mL^−1^	UHPLC-MS/MS	Amino acid	ROS generation	92
	[20–30 × 100–200]			72 h		Lipid	Oxidative stress	
						Purines, and pyrimidines	Inflammation	
							Apoptosis	
Fe_2_O_3_	[16–44 × 45–173]	N/A	THP-1	20–100 μg mL^−1^	LC-ToF-MS	Sphingolipids	Inflammation	272
	[20–53 × 88–322]			24 h		Ceramides	Energy metabolism disruption	
						Sphingosine-1-phosphates	Cell proliferation	
						Glucosylceramide	Autophagy	
Fe_3_O_4_ FeO_*x*_ mix	19.4	N/A	BEAS-2B	0.003, 0.03, 0.3 μg mL^−1^ 24 h	DI-MS	No significant change	Low cytotoxicity	273
	10.6, 5.5							
ZrO_2_	31.9	N/A	MC3T3-E1	100 μg mL^−1^	LC-ToF-MS	Phospholipids ↓	Oxidative stress	274
				24 h				
				48 h				
MoS_2_–Ag	Ag – 18	Chitosan	Yeast *S. cerevisiae*	20 μg L^−1^ N–Ag, 1 mg L^−1^ CS–MoS_2_	GC-TOF-MS	Amino acid	Oxidative stress lower than Ag alone	275
				24 h		Organic acid	Membrane stress more than Ag alone	
						Fatty acid		
MoS_2_	5–10	N/A	Mesophyll protoplasts	50 μg mL^−1^	GC-MS	ATP ↓	ROS generation	276
				0.5–3 h		3-(4-Hydroxyphenyl) propionic acid ↑	Mitochondrial dysfunction	
						Catechin ↓	Photosynthesis decrease	
MoS_2_	N/A	N/A	*E. coli*	1, 10, 100, 1000 μg mL^−1^	GC-TOF-MS	Glycine, serine, and threonine	Antimicrobial activity	277
				6 h		Urea cycle	LDH release	
						Pyruvate	ROS generation	
MoS_2_	Thickness – 4	Chitosan	HDFs	25, 100 μg mL^−1^	GC-TOF-MS	Amino acid	Membrane damage	278
				72 h		GSH ↓	ROS generation	
						TCA ↓	DNA damage	
							Inflammation	
							Apoptosis	
WS_2_	100–4000	N/A	A549 – THP-1	50, 100 μg mL^−1^	GC-MS	Amino acid	Bystander effect	279
				24 h		Glycolysis ↑	Energy metabolism disruption	
						Pyruvate	Phagocytosis	
							Cell migration	
Fe	50	N/A	Mesophyll protoplasts	50 μg mL^−1^	GC-MS	ATP ↑	Photosynthesis enhancement	276
				0.5–3 h		Phenylalanine ↓		
Fe	6.6	N/A	*S. aureus*	100 μg mL^−1^	^1^H NMR	α-Linolenic acid	No significant effect on bacterial metabolism	280
			*E. coli*	0.5 h	UHPLC-HRMS	Lipoic acid metabolism		
						Phenylalanine		
						Tyrosine, and tryptophan biosynthesis		
Bismuth	<100	Carboxyl	*H. pylori*	100 μg mL^−1^	^1^H NMR	TCA	Antimicrobial activity	281
				24 h		Nucleotide	Release of some metabolites to the supernatant	
						Amino acid		
Cu	5.3	N/A	*S. aureus*	100 μg mL^−1^	^1^H NMR	Pentose phosphate	Blocking cell metabolism	280
			*E. coli*	0.5 h	UHPLC-HRMS	Amino sugar		
						Nucleotide sugar		
Cu(OH)_2_	140	N/A	HepG2	25 μg mL^−1^	UHPLC-MS/MS	Glycolysis ↑	Energetic stress	282
				48 h		Lipid ↑	Oxidative stress	
						TCA ↓		
S (α- or β-SNPs)	10	PEG	*A. niger* (MTCC-10180)	4 mg mL^−1^	GC-MS	TCA ↓	Antimicrobial activity	283
	50			16 mg mL^−1^			Oxidative phosphorylation	
				48 h				
Cu–Fe	15	N/A	*S. aureus*	100 μg mL^−1^	^1^H NMR	Pentose phosphate	Blocking cell metabolism	280
			*E. coli*	0.5 h	UHPLC-HRMS	TCA	Cell death	
						Amino sugar		
						Nucleotide sugar		
Au–Pd	43.8	N/A	HUVECs	8, 80 μg mL^−1^	GC-MS	Glycolysis ↑	Mitochondrial dysfunction	284
				48 h		Pentose phosphate ↑		
						Lipid ↑		
						TCA ↓		
Ti_3_C_2_	489 × 11	N/A	HUVEC	100, 200, 500 μg mL^−1^	GC-MS	TCA ↓	Energy metabolism disruption	285
				48 h	LC-MS/MS	Glycolysis ↑		
						Fatty acid ↑		
						Lipid ↑		

### 5.7 Carbon-based NPs

#### 5.7.1 Graphene

Graphene has attracted significant attention due to its unique and novel properties, which has promising applications in different fields, including biomedical engineering, tissue engineering, and biosensors. However, graphene-based drug delivery systems and other biomedical applications are associated with challenges related to the safety of carbon NMs for clinical use. Many groups have investigated the cytotoxicity of graphene. In this case, although the conventional *in vitro* toxicity assays of graphene yielded contradictory results, Jiao *et al.*^286^ used the metabolomics approach to investigate the metabolic responses on graphene-treated HepG2 and detected twelve metabolites as potential biomarkers. The authors also determined three KEGG pathways including arginine and proline metabolism, purine metabolism, and glycophospholipid metabolism.

Adamson *et al.*^287^ studied the metabolic change caused in macrophages by graphene nanoplatelets. The number of compounds changed following exposure to graphene was determined to be both concentration and time dependent. The identified metabolites are related to several metabolism pathways, such as GSH metabolism, pantothenate and CoA biosynthesis, sphingolipid metabolism, purine metabolism, arachidonic acid metabolism and others. Graphene oxide (GO) also has some biomedical applications but a greater understanding of its cytotoxicity and efficiency as a drug carrier is needed. Raja *et al.*^288^ used NMR-based metabolomics to assess the metabolic effect of GO nanosheets on MCF-7 breast cancer cells. The treatment affected arginine metabolism, proline metabolism, and aminoacyl-tRNA biosynthesis, including anabolism and catabolism. Moreover, GO increased the number of metabolic disturbances in cancer steroids in a dose-dependent manner.

#### 5.7.2 Carbon black NPs (CBNPs)

Carbon black NPs (CBNPs) are the core component of fine particulate matter in the atmosphere, which make its exposure to the respiratory system easy. It was reported that CBNPs can induce inflammation, oxidative stress, and changes in cell signalling and gene expression in mammalian cells and organs. Hou *et al.*^289^ used MS-based metabolomics to reveal this mechanism in A549 cells. Their study identified a total of 32 differential metabolites between the CBNP exposure and control groups. The pathway analysis showed that the metabolic changes were involved in tricarboxylic acid (TCA) cycle, alanine, aspartate, glutamate, and histidine metabolism. This suggests that CBNPs act by affecting the normal process of energy metabolism and disturbing several vital signalling pathways in the cells, finally leading to cell apoptosis. Other studies performed *in vivo* experiments and assessed the effect of carbon-based NMs on the ecosystem by studying some models such as earthworms. For instance, Xu *et al.*^290^ studied the impacts of three carbon NMs, *i.e.*, carbon black (CB), reduced graphene oxide (RGO), and single-wall carbon nanotubes (SWCNTs), on *Eisenia fetida*, an early warning soil invertebrate for pollution events. They concluded that the soil environmental risk of C-NMs was related to their particle morphology, contributing to a comprehensive understanding of nano-agriculture. Table 8 summarizes the studies that used the metabolomics technique to assess the effect of other C-based NPs *in vitro* on different cells.

**Table 8:** Summary of C-based NP-induced perturbation of metabolic pathways and their biological impact on different cells

NP	Size [nm]	Coating	Cell	Dose/exposure time	Analytical platform	Perturbed metabolic pathway	Biological effect	Ref.
G	N/A	N/A	HepG2	25 μg mL^−1^	UPLC-MS	Arginine and proline	Membrane damage	286
						Purine	Protein damage	
						Glycophospholipid		
						Urea cycle		
G	2000 × 2000 × 12	N/A	RAW 264.7	50, 100 μg mL^−1^	HPLC-MS	GSH	Mitochondrial membrane potential	287
				1, 3 h		Sphingolipid	CD36-dependent response	
						CoA		
						Purine		
G	N/A	N/A	HaCaT	5 μg mL^−1^	^1^H NMR	Fumarate ↑	ROS generation	291
				168 h		Glycerophosphocholine ↑	Mitochondrial damage	
						Pyruvate ↓	Cell death	
						Phosphocreatine ↓	Cell migration	
						Phosphocholine ↓		
G	N/A	Carboxyl	A549	0.01, 0.1 μg mL^−1^	LC-MS	Amino acid	ROS generation	292
				48 h		Organic acids	Apoptosis	
						Glycerophospholipid		
						Glycerolipids		
GO	N/A	N/A	HaCaT	5 μg mL^−1^	^1^H NMR	Fumarate ↓	ROS generation	291
				168 h		Alanine ↑	Mitochondrial damage	
						Pyruvate ↑	Cell death	
						Phosphocreatine ↑	Cell migration	
						Glycerophosphocholine ↑		
GO	N/A	N/A	MCF-7	20, 40, 60 μg mL^−1^	^1^H NMR	Arginine	Catabolism	288
				24 h		Proline	Therapeutic activity	
						GSH		
						TCA		
GO	300	PEG	Saos-2	75 μg mL^−1^	^1^H NMR	Amino acid ↓	Proliferation delay	293
				24 h		Taurine ↓	Oxidative stress	
						Creatine ↓		
						Phosphocholine ↑		
						Nucleotide ↑		
GO	N/A	N/A	RLE-6TN	10 μg cm^−2^	HPLC-MS/MS	Amino acid	Oxidative stress	231
				24 h	FIA-MS/MS	Biogenic amine	Apoptosis	
						Lipid		
GO	N/A	N/A	NR8383	2.5, 5, 10 μg cm^−2^	HPLC-MS	No significant alteration	Oxidative stress	243
				24 h			Mitochondrial dysfunction	
							DNA damage	
							Cell death	
GO	500–5000 × 0.8–1.2	N/A	Alga *C. vulgaris*	0.01–10 μg mL^−1^	GC-MS	Alkanes, lysine, octadecadienoic acid and valine	ROS generation	294
				96 h			Oxidative stress	
MWCNT	500–2000 × 8–15 × 3–5	None	BEAS-2B	2–39 μg mL^−1^	^1^H NMR	Choline	Oxidative stress	295
		Hydroxyl	HepG2	24 h		Betaine	Inflammation	
		Carboxyl				Succinate	Profibrosis	
							DNA damage	
MWCNT	[(1–3) or (0.05–0.2) 10^4^ × 20–30 or 8–15]	Hydroxyl	BEAS-2B	6–45 μg mL^−1^	^1^H NMR	TCA	Oxidative stress	296
			HepG2	24 h		GSH	Inflammation	
						Amino acid	Profibrosis	
							DNA damage	
MWCNT	846 × 11	N/A	HBEC-3KT	0.96, 1.92 μg cm^−2^	FT-MS/MS	TG, SM, Cer, PE, Chol ↑	ROS generation	297
				2, 13 weeks			Apoptosis	
SWCNT	500–3000 × 1–2	Carboxyl	Alga *C. vulgaris*	0.01–10 μg mL^−1^	GC-MS	Alkanes, lysine, octadecadienoic acid and valine	ROS generation	294
				96 h			Oxidative stress	
CNT	N/A	Carboxyl	A549	0.01, 0.1 μg mL^−1^	LC-MS	Amino acid	Energy metabolism dysfunction	292
				48 h		Organic acids	ROS generation	
						Glycerophospholipid	Apoptosis	
						Glycerolipids		
C_60_	100	Carboxyl	A549	0.01, 0.1 μg mL^−1^	LC-MS	Amino acid	Energy metabolism dysfunction	292
				48 h		Organic acids		
						Glycerophospholipid		
						Glycerolipids		
C_60_	120–150	N/A	Alga *S. obliquus*	0.1, 1 μg mL^−1^	GC-Q-TOF-MS	TCA	Growth inhibition	298
				168 h		Sucrose, d-glucose, malic acid ↑	Photosynthesis decrease	
C_black_	30–50	N/A	A549	70 μg mL^−1^	UHPLC-Q-TOF-MS	TCA ↑	Inflammation	289
				48 h		Alanine	Oxidative stress	
						Aspartate	Energy and signalling pathway disruption	
						Glutamate	Apoptosis	
						Histidine ↑		
C_black_	14	None	EA.hy926	0.001–1 μg mL^−1^	GC-MS	Amino acid	Enhanced cell proliferation	299
		Benzo[*a*]pyrene		72 h		Organic acid	Cell motility	
						Fatty acid	Cell migration increase	
C_cellulose_	2–40	N/A	NG97	10, 100 μg mL^−1^	LC-MS	Fatty acid	Cell viability	300
				6 h			Metabolic activity disruption	
Cyclodextrin	0.5–1	Folate	MDA-MB-231	1 : 16 (v/v)	LC-Q-TOF-MS	Hexose metabolism	Apoptosis	301
				24 h		Glycolysis		
						TCA		
Glycyrrhizin (GL-BSA-Lut-NPs)	225.3	Luteolin	Bel-7402	0.375, 0.75, 1.25, 2.5, 5 mg mL^−1^	^1^H NMR	Lactate, 3-hydroxybutyrate, lipid, tyrosine, and β-glucose↓	Antitumor effect	302
		BSA		24 h		Glutamate, asparagine, choline, and creatine ↑	Apoptosis	

### 5.8 Polymeric NPs

Polymeric NPs such as polystyrene (PS) are gaining considerable attention because of their growing accumulation in the environment and the high probability of human and animal exposure. Therefore, more research must be done to increase our understanding of their potential effects. Kim *et al.*^303^ studied the metabolic effects of PS NPs on human epithelial colorectal cells (Caco-2). The authors designed two methods to investigate the exposure of Caco-2 cells to NPs, where the first is by exposing cells to a high concentration of 50 nm PS NPs for 24 h (acute), and the second is by exposing them to a relatively lower concentration for over 1.5 months (chronic). The biological assays were performed using specific NP concentrations, which were 10 and 80 μg mL^−1^ for the acute model and 0.1 μg mL^−1^ for the chronic exposure model. After acute exposure, untargeted metabolic profiling was performed and the change in lipid metabolic pathways determined, including steroid and arachidonic acid metabolism. Alternatively, chronic exposure induced relatively minor changes. However, there was still a potential effect on fatty acid biosynthesis, indicating that acute and chronic exposure to PS NPs may disturb lipid homeostasis. They also confirmed the changes in the expression levels of lipid transcriptional regulatory factor coding genes, namely, sterol regulatory element-binding transcription factors 1 and 2. The total fatty acid composition was further studied to verify metabolic disturbance by chronic exposure. Su *et al.*^304^ investigated the effect of poly(l-lactic acid) (PLLA) nanofibers on PC12 cell differentiation at the metabolic level. Many differential metabolites were identified and two pathways and three metabolites critical to PC12 cell differentiation were influenced by the nanofibers. Table 9 summarizes the studies that used the metabolomics technique to assess the effect of other polymeric NPs *in vitro* on different cells.

**Table 9:** Summary of polymeric NP-induced perturbation of metabolic pathways and their biological impact on different cells

NP	Size [nm]	Coating	Cell	Dose/exposure time	Analytical platform	Perturbed metabolic pathway	Biological effect	Ref.
PS	72	N/A	BEAS-2B	10, 50 μg mL^−1^	HPLC-MS/MS	Amino acid ↑	Anti-oxidative protection	247
				24 h	GC-MS/MS	TCA ↑	Autophagy	
						Lactate ↑	Perturbed energetic metabolism	
PS	50	N/A	Caco-2	Acute [10, 80 μg mL^−1^ – 24 h]	UPLC-MS	Acute – lipid; steroid and arachidonic acid	Metabolic disturbance	303
				Chronic [0.1 μg mL^−1^ – 45 days]		Chronic – fatty acid biosynthesis	ROS generation	
PS	50	Amino	Cyanobacterium *S. elongatus*	2.5, 4 μg mL^−1^	GC-ToF-MS	Amino acid ↓	Oxidative stress	305
				48 h		GSH ↓	Membrane damage	
						*o*-Phosphoethanolamine ↑	ROS generation	
PLGA	100–120	None	A549	92 μg mL^−1^	LC-MS	Nucleotide	Energy metabolism disruption	306
		MTX	THP-1	24 h		TCA ↓	More effective on cancer cell than free MTX	
						Glycolysis ↓		
						GSH ↓		
PLGA	100–125	N/A	RAW 264.7	10, 500 μg mL^−1^	^1^H NMR	Glycolysis ↑	Inflammation	244
				24, 48, 72 h		Lactate ↑	Energy metabolism disruption	
PLGA	250.90	l-Carnitine	CFs	2 mg mL^−1^	GC-MS	Amino acid ↑	Very effective drug carrier for amino acid metabolism	307
				144 h		2-Ketoisocaproic ↑		
						Glucose ↑		
PLLA	246.71	N/A	PC12	N/A	MQ-ToF-MS	Amino acid	Cell differentiation enhancement	304
	218.57			12, 24, 36 h	UHPLC-MS	Carbohydrate		
						Lipid		
PLLA (LJ@AA)	150	PEG–amino acid	MCF7	1 μg mL^−1^	UPLC-MS/MS	Amino acid ↓	Cancer inhibition	157
				48 h				
PET	10–80	N/A	Caco-2	30 μg mL^−1^	^1^H NMR	Glucose ↓	Oxidative stress	308
				48 h		Lactate ↓		
						Alanine ↑		
Silk	10	N/A	RAW 264.7	10, 500 μg mL^−1^	^1^H NMR	Glycolysis ↑	Inflammation	244
	0–125			24, 48, 72 h		Lactate ↑		
						Pyruvate ↓		
Strigol1/albumin/chitosan	5–10	N/A	HepG2	9.24–92.4 nM	LC-MS-MS	Spermine and spermidine ↑	Apoptosis	309
				48 h		Glutamine ↓	Anti-carcinogenic effect	
						Fumarate ↓		
Platicur-NCs	100	Chitosan	HeLa	Dark – 75 μM	^1^H NMR	Light – glutamine, acetate, glucose ↑	ROS generation	310
		Chitosan–pectin		Light – 0.05 μM		Dark – lactate, creatine, glycine, choline ↑	Oxidative stress	
				24 h		GSH ↓	Apoptosis	
6OCaproβ	15	N/A	MCF-7	N/A	Q-TOF-LC	Serine biosynthesis	Apoptosis	311
Cyclodextrin	3			48 h	MS	Estrogen biosynthesis		
						Phospholipid biosynthesis		
Lipo-lysosomes	97.88	l-Carnitine	CFs	2 mg mL^−1^	GC-MS	Amino acid ↑	Very effective drug carrier for the synthesis of unsaturated fatty acids	307
				144 h		2-Ketoisocaproic ↓		
						3-Phenyllactic acid ↓		
						2-Hydroxybutyrate ↑		
Cationic liposomes	15	N/A	L02	116 μg mL^−1^	UHPLC-Q-TOF-MS	Sphingolipid	ROS generation	312
	2.2			24 h		Fatty acid	Oxidative stress	
						TCA	Energy metabolism disruption	
						GSH		
						Methionine		
						Pyrimidine		
Chloroquine	23	Curcumin	Parasite *P. falciparum*	0.1–2.5 μg mL^−1^	^1^H NMR	Pyrimidine	Effective drug carrier	313
Dendrimer	9			48 h		TCA ↑		
						GSH ↑		
Lambda phage like	16	Fluorescein-5-maleimide	SKBR3	150 nM	UHPLC-MS	TCA ↓	DNA damage	314
		Trastuzumab		2 μM		Glycolysis	Oxidative stress	
				3 h		Fatty acid synthesis	Mitochondrial dysfunction	
						Protein synthesis		

## 6. Conclusions

Currently, it is obvious for many researchers that nanotechnology provides countless benefits, and consequently its demand is increasing daily. It is very important to assess the safety of every NP that is being produced and to test its beneficial and disadvantageous effects. Understanding the interactions between NPs with cells and how NPs are internalized in cells are the first step to assess their toxicity. Here, not only the type of the NP matters, but also its physicochemical properties such as size, shape, and surface properties. It was proven that NPs with different properties have different effect on cells. Some sizes of NPs are not toxic, but others are severely harmful to cells. In general, conventional assays are the most used strategy to assess the effect of NPs on cells. However, these assays were found to interfere with NPs, giving false results in some cases, and they are unable to reveal the molecular information of the toxicity or effect of NPs. Thus, an increasing number of researchers are heading towards the use of other analytical techniques. Metabolomics is a powerful technique that provides a full picture of the toxicity of NPs by analyzing the functionality of an existing living system by measuring the metabolic change induced by NPs. Unlike conventional assays, this tool does not interfere with NPs and provides information at the molecular level about the toxicity of NPs. Furthermore, it forms an additional bridge that connects the *in vitro* with the *in vivo* models, as proven by several references. It was shown in this review that NPs can harm the cell through different ways, including cell viability and proliferation perturbation, inflammatory response, oxidative stress, ROS generation, and cell death *via* apoptosis or necrosis. Moreover, using metabolomics, NPs were shown to perturb the metabolic pathways of cells, including the TCA cycle, DNA and protein synthesis, glycolysis, glutathione, and amino acid pathways. Thus far, metabolomics has been used in many studies to assess the effects of different NPs on living organisms. However, more research needs to be done to identify and validate specific biomarkers of the effects of NPs on cells. Reaching this point will introduce a huge step in determining the toxicity of NPs and how to avoid or multiply this toxicity. This will help in designing better NPs for biomedical applications and producing safer NPs for industrial applications. Nevertheless, long-term targeted studies should also be performed to fill many gaps in this field. Also, the combination of metabolomics with other techniques is required in some cases to provide a bigger picture on the events occurring in the cell.

## Author contributions

Mohammad Awashra: literature search, writing, figures designing and creating. Piotr Mlynarz: review idea, reviewing, and editing.

## Conflicts of interest

There are no conflicts to declare.

## Supplementary Material
